# Cataloguing and Theorising Open Research Practices in the Arts, Humanities and Social Sciences: Problematising and Diversifying ‘Open Science’

**DOI:** 10.12688/f1000research.178353.2

**Published:** 2026-08-20

**Authors:** Jenni Adams, Miranda Barnes, Samuel Moore, Stephen Pinfield

**Affiliations:** 1 University of Sheffield School of Information, Journalism and Communication, Sheffield, UK; 2 Cambridge University Library, Cambridge, UK; 3 Cambridge Digital Humanities, Cambridge, UK

**Keywords:** open research, Open Science, humanities, social sciences, metaresearch

## Abstract

**Background:**

Discourses of ‘Open Science’ have been criticised for privileging the quantitative and positivist methodologies of science, technology, engineering and mathematics (STEM) research at the expense of qualitative, interpretive, critical-theoretical, practice-based, and other forms of research more common in the arts, humanities and social sciences (AHSS).

**Methods:**

In this article, which emerged from a work component of the MORPHSS (Materialising Open Research Practices in the Humanities and Social Sciences) project, we document the process, outcomes and practical implications of work to develop a catalogue of open research practices in these disciplines, which proceeded via a wide-ranging literature review followed by targeted desk research and a conceptual mapping exercise.

**Results:**

Key findings include the fact that open research practices in AHSS are diverse, extending beyond the suite of practices emphasised within dominant accounts of Open Science. We identify among these practices a range of forms of openness including those focused on mobilising the involvement and expert knowledge of diverse participants and communities.

**Conclusions:**

Presenting a typology of forms of openness in AHSS that is consistent with the epistemic logics of these disciplines, we conclude that openness in AHSS is highly situated and context-dependent, as well as resisting quantification and binary measurement. Drawing on these conclusions, we offer a series of recommendations for institutions, open research monitoring initiatives, funders, publishers, learned societies and researchers to enhance the inclusivity of their policy and practice around openness.

## Introduction

Discourses of ‘Open Science’ (OS) have been criticised for their ‘narrow epistemologies’ ( Ross-Hellauer et al., 2022, 16) which privilege the quantitative and positivist methodologies of science, technology, engineering and mathematics (STEM) research at the expense of qualitative, interpretive, critical-theoretical, practice-based, and other forms of research more common in the arts, humanities and qualitative social sciences. Where the presence of OS practices such as pre-registration and open data and code is used as an evaluative measure, this consequently carries the risk of marginalising and undervaluing the work of arts, humanities and social sciences (AHSS) scholars relative to peers in STEM on the basis of an inappropriate and epistemically incongruent set of criteria. This phenomenon, which Leonelli (2023, 39) considers an instance of epistemic injustice, has prompted calls to reframe open research in ways more consistent with AHSS practices, most notably in calls for an ‘open humanities’ ( Arthur and Hearn, 2024; Knöchelmann, 2019) and emerging advocacy around open qualitative research (e.g. Prosser et al., 2023; Steltenpohl et al., 2025). Nevertheless, what remains to take place is a systematic, wide-ranging effort to survey and document a broad array of open practices across AHSS disciplines and to theorise the forms of openness these embody. In this article, which reports work undertaken as part of the Materialising Open Research Practices in the Humanities and Social Sciences (MORPHSS) project, we document the process and outcomes of efforts to achieve this by creating a catalogue of open research practices in AHSS. We highlight these practices’ manifestations of forms of openness that are under-emphasised in the dominant discourse of Open Science and present a series of recommendations for institutions, open research monitoring initiatives, funders, publishers, learned societies and researchers to enhance the inclusivity of their policy and practice around openness.

## Background

### Problematising ‘Open Science’

The term ‘open research’ (OR) ^1^ refers to practices aimed at making the processes and outputs of research more widely available. Its aims and purposes include addressing knowledge inequities; facilitating collaboration to address social and scientific problems; democratising the research process by reimagining the boundaries between the academy and society; and enabling enhanced scrutiny of findings ( Beigel, 2024, 1; Cole et al., 2023, 2; Munafò, 2016). The 2021 UNESCO Recommendation on Open Science reflects this diversity of aims and mechanisms:

[O]pen science is defined as an inclusive construct that combines various movements and practices aiming to make multilingual scientific knowledge openly available, accessible and reusable for everyone, to increase scientific collaborations and sharing of information for the benefits of science and society, and to open the processes of scientific knowledge creation, evaluation and communication to societal actors beyond the traditional scientific community. It comprises all scientific disciplines and aspects of scholarly practices, including basic and applied sciences, natural and social sciences and the humanities, and it builds on the following key pillars: open scientific knowledge, open science infrastructures, science communication, open engagement of societal actors and open dialogue with other knowledge systems. (2021, II.6)

Nevertheless, the concept of ‘Open Science’ (OS) as it is mobilised in practice, especially in Global North contexts, fails to align with this inclusive vision, instead placing emphasis largely on the pillars of ‘open scientific knowledge’ and ‘open science infrastructures’ and on the purpose of reproducibility as a vehicle for research reform. As Albornoz et al. note:

Most mainstream narratives about Open Science, emerging particularly from Europe and North America, envision open science as a system of technology-driven tools and processes that, when utilised, are assumed to accelerate scientific discoveries, improve transparency and reproducibility of research […] and improve accountability to the scientific community as well as to the public. (2017, 294)

This efficiency-driven, reproducibility-focused approach ^2^ to openness fails to address global inequities in access and participation, often exacerbating inequalities ( Dutta et al., 2021, 807; Hillyer et al., 2017). Moreover, its emphasis on the availability of a standardised set of outputs as a means to verify and incentivise reproducibility prioritises the quantitative and positivist forms of inquiry within which these outputs and aims are methodologically congruent above other forms of knowledge creation – humanistic, qualitative, critical-theoretical, archival, arts-based, to name but a few. As Steltenpohl et al. (2023) note, ‘[w]ithin the open science movement, discussions around rigor and transparency have largely come from a positivist, quantitative perspective that focuses on the transparency of outputs, namely open data, open materials, open code, and open access to manuscripts’, with the result that ‘[o]pen science guidelines fail to account for research based on epistemologies that are not strictly positivist and methods that are not strictly quantitative in nature’ (n.p., n.p.).

This would be less problematic were such expectations not universalised as a default model of openness applicable to all forms of research ( Bazzoli, 2022). Such universalisation is evident in national and funder open research policies, especially in the Global North, which tend to focus largely on open access and open data at the expense of other open practices ( Albornoz et al., 2018; for examples, see Bulock, 2023; Murphy and Thomas, 2023; UKRI, 2025). The centring of open data suggests a focus firstly on practices that support reproducibility/replicability, and secondly on disciplines for which this practice is more straightforward and achievable, while conventional framings of open access stipulations likewise foreground STEM research. Publications are assumed to be journal articles emerging from funded projects, where payment of article processing charges (APCs) is both established and feasible. The need to enable open circulation of knowledge produced in AHSS projects – often unfunded and resulting in monographs – is underemphasised. The exclusion of broader dimensions of open research such as the open engagement of societal actors and knowledge systems is further underscored by the findings of a 2025 study of EDI and public participation in Open Science policy documents across Europe and the Americas ( Chtena et al., 2025), which noted that Open Science policies prioritise the accessibility of outputs over aspects of openness aimed at equity and inclusion. If AHSS disciplines are covered in such policy approaches, there is often an assumption that they possess straightforward equivalents to the data, publications and other outputs created in STEM and are able to share these in comparable ways.

Such universalisation of expectations is also evident in Open Science guidance and advocacy materials such as the apparently discipline-neutral TOP guidelines, which aim to inform journal policy on openness yet focus primarily on transparency around materials, data and code. ^3^ The Center for Open Science, the organisation behind TOP, also provides a discipline-neutral Open Science Badges initiative that encourages journals to recognise authors for use of just three open practices: pre-registration, open data and open materials. Given the precarious and underfunded nature of AHSS research, especially in the humanities, the positivist and quantitative orientation of dominant conceptions of OS risks exacerbating existing knowledge hierarchies and tangibly disadvantaging scholars in the arts, humanities and qualitative social sciences through their subjection to inappropriate forms of evaluation ( Penders et al., 2019, 2; Ross-Hellauer et al., 2022, 16; Ulpts, 2024, 2).

The disjunction between the framing of Open Science expectations as universally applicable and their uneven relevance across a diverse range of research practices might be one reason for what are often regarded as limited levels of engagement with open research across the humanities and social sciences (e.g. Broadhead and Gonnet, 2025; Ferreira and Borges, 2022; Hardwicke et al., 2020; Jeng and He, 2022; Quigley, 2021; Robinson-Garcia et al., 2020; Severin et al., 2018). In requiring adherence to stipulations and goals that may lack relevance to their knowledge practices, and in implicitly denying the legitimacy of forms of knowledge work outside these frameworks, OS discourse risks alienating researchers in the arts, humanities and qualitative social sciences, potentially to the extent of discouraging engagement with the broader possibilities of open research.

### Introducing MORPHSS

The Materialising Open Research Practices in the Humanities and Social Sciences (MORPHSS) project sets out to address precisely this under-recognition of forms of openness appropriate to AHSS research within dominant OS paradigms. Such work could be framed in a variety of different ways – e.g. through a focus on methodologies, epistemic cultures ( Knorr-Cetina, 1999) or Leonelli’s more granular ‘systems of practice’ ( 2023, 31). However, we frame the project in disciplinary terms for pragmatic reasons: disciplinary categories are central in the circulatory and regulatory mechanisms of research, as well as accommodating a diversity of practice we would not wish to overlook in a focus on, for example, interpretive or qualitative approaches. We nevertheless acknowledge that ‘AHSS’ includes some areas of research – namely, the quantitative social sciences and computational digital humanities – that are already well-catered for within dominant OS paradigms. These areas are consequently less emphasised within this work. In terms of the component disciplines of AHSS, we drew in our literature searches (below) on the UK’s Research Excellence Framework (REF) list of units of assessment for the arts, humanities and social sciences, ^4^ though in practice we recognise some areas of fluidity around disciplines that are included (e.g. geography, which in some instances might be either a social or physical science) and excluded (e.g. psychology and public health, qualitative work in which has experienced similar challenges in terms of misalignment with quantitative OS discourses). ^5^ It is also important to recognise considerable variation within disciplines.

The present article documents the process and outcomes of work undertaken by MORPHSS to catalogue open research practices in the arts, humanities and (qualitative) social sciences. In this project component, we sought to map areas of traction of and resistance to existing OS discourses within AHSS disciplines, with a particular focus on identifying and cataloguing open practices appropriate to AHSS methodologies. Our research questions were:

• **Where, how, and how successfully** are existing discourses of ‘open research’ / ‘Open Science’ applied to research practices in the arts, humanities and social sciences? (RQ1)
• **How and from what perspectives is such an application resisted?** (RQ2)
• **What existing, emergent and experimental open research practices** are taking place in the arts, humanities and social sciences? (RQ3)
  ○ What *forms* of openness exist in AHSS?
  ○ How are these specific to the research types and disciplines concerned? How can these add plurality and diversity to our broader concepts of ‘openness’ in research?

Exploring these questions via a literature review followed by targeted research, we sought to create a catalogue of open AHSS practices that can be used as a starting point in ongoing explorations of openness in these areas. This article documents and reflects on the catalogue's creation before examining the forms and practices of openness we identified.

## Methodology

The core research team for this project comprised researchers and professionals at research-intensive universities in the UK. Our areas of expertise are scholarly communications and metaresearch, literature and creative writing, publishing, digital humanities and information science. Our methodological approaches include qualitative, mixed-methods, critical-theoretical and arts-practice research.

We sought an entry point onto the intersection of research openness and AHSS via a literature review that combined systematic search strategies with more exploratory approaches. These processes are detailed in full in our openly available Literature Review Protocol ( Adams et al., 2026b); a summary is below.

### Systematic search strategies

We searched the following bibliographic databases for academic articles, monographs, edited collections, preprints, conference proceedings and grey literature: Scopus, Web of Science, Dimensions, Humanities Index, MLA International Bibliography, ASSIA (Applied Social Sciences Index and Abstracts); LISA (Library and Information Science Abstracts) and Overton, in addition to the preprint repositories OSF Preprints, SSRN and preprints.org. Searches targeted the co-occurence of terms associated with open research and disciplinary signifiers, with the latter considered at both macro (e.g. humanities, social sciences, AHSS, HASS, HSS) and more granular levels. Terms relating to specific disciplines (e.g. linguistics, education, social work) were drawn from the UK’s REF list of units of assessment for the arts, humanities and social sciences. An overview of the search terms used can be found in Table 1; further information on relevance criteria is available in the Literature Review Protocol. Terms from the vocabulary of Open Science were included to assess its traction in AHSS as well as capturing instances of oppositional reframing. Terms relating to open access were not included in order to focus primarily on areas of open practice in AHSS that are not already well-documented. ^6^ While the scope of material sought was not limited by geographical area, it was restricted to the English language to reflect the research team’s expertise.

**Table 1.** Overview of key systematic searches.

Key search terms (adapted to each individual database)
Title “Open research” OR “open science” OR reproducib* OR “open data” OR “data sharing” OR “open software” OR “open code” OR “open practice” or “open practise” OR preregistration OR pre-registration OR preprint* AND Humanities OR “Social Sciences” OR “AHSS” OR “HSS” OR “HASS” OR [specific disciplines, terms taken from REF Units of Assessment] Parameters: 2010<; disciplinary parameters (AHSS disciplines) where available.
Title – abstract – keywords “Open humanities” OR “open social sciences” Parameters: 2010<; disciplinary parameters (AHSS disciplines) where available.
Title – abstract – keywords – full text “Open research” AND (method* OR practice*) AND Humanities OR “social sciences” or “AHSS” OR “HSS” OR “HASS” OR [specific disciplines, terms taken from REF Units of Assessment]) Parameters: 2010<; disciplinary parameters (AHSS disciplines) where available; proximity operators (w/3) for the co-occurence of the first two components where available.
Title – abstract – keywords “Open research” OR “open science” OR reproducib* OR replica* OR transparen* AND “Epistemic diversity” OR “epistemic plural*”

Legend: Details of the key systematic searches that were performed.

### Exploratory strategies

Acknowledging the limitations of systematic search strategies alone ( Greenhalgh and Peacock, 2005), especially in the context of potentially emergent practices and approaches which may not be explicitly framed within a vocabulary of open research, we coupled these searches with a series of more exploratory approaches. Specific exploratory strategies included iterative/evolving search strategies, citation exploration, targeted engagement with sources of different media types (e.g. podcasts, film) and serendipitous discovery. These strategies also included selecting a small number of journals focused on qualitative/AHSS methods, digital publishing and digital preservation and scanning the contents of issues over the last 10 years for relevant material. ^7^ They also included direct searches for grey literature by organisation, including UNESCO, publishers and funders, UKRN, RDA, OASPA, ALPSP, JISC, DARIAH, OPERAS, FAIRsharing, Open Access Tracking Project ( OATP), Science Europe and more.

### Sifting, evaluation and annotation process

**Figure 1.**
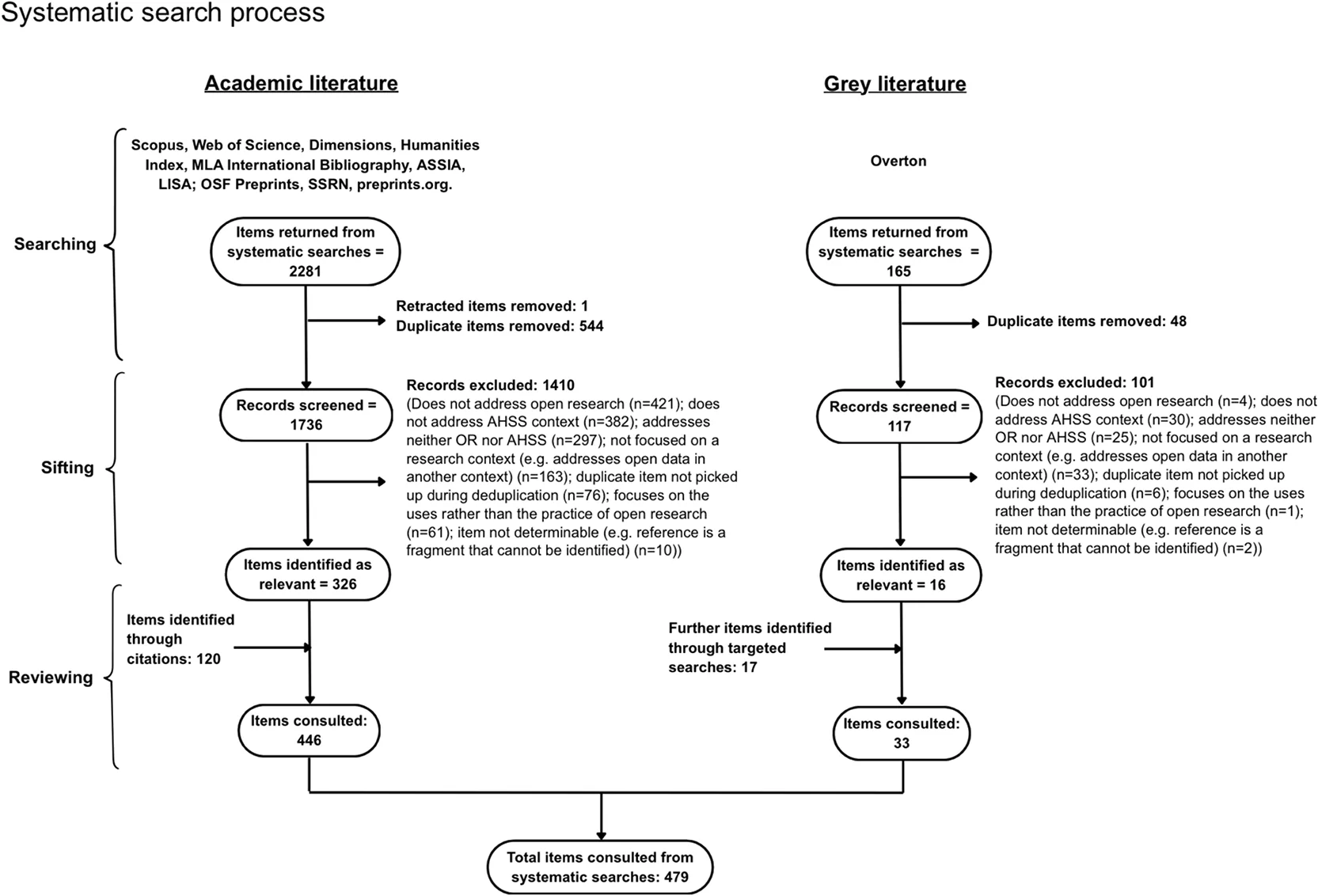
Flow diagram of systematic search process. Legend: The figure details the number of academic and grey literature sources identified, screened and reviewed as part of the systematic search process for this study.

**Figure 2.**
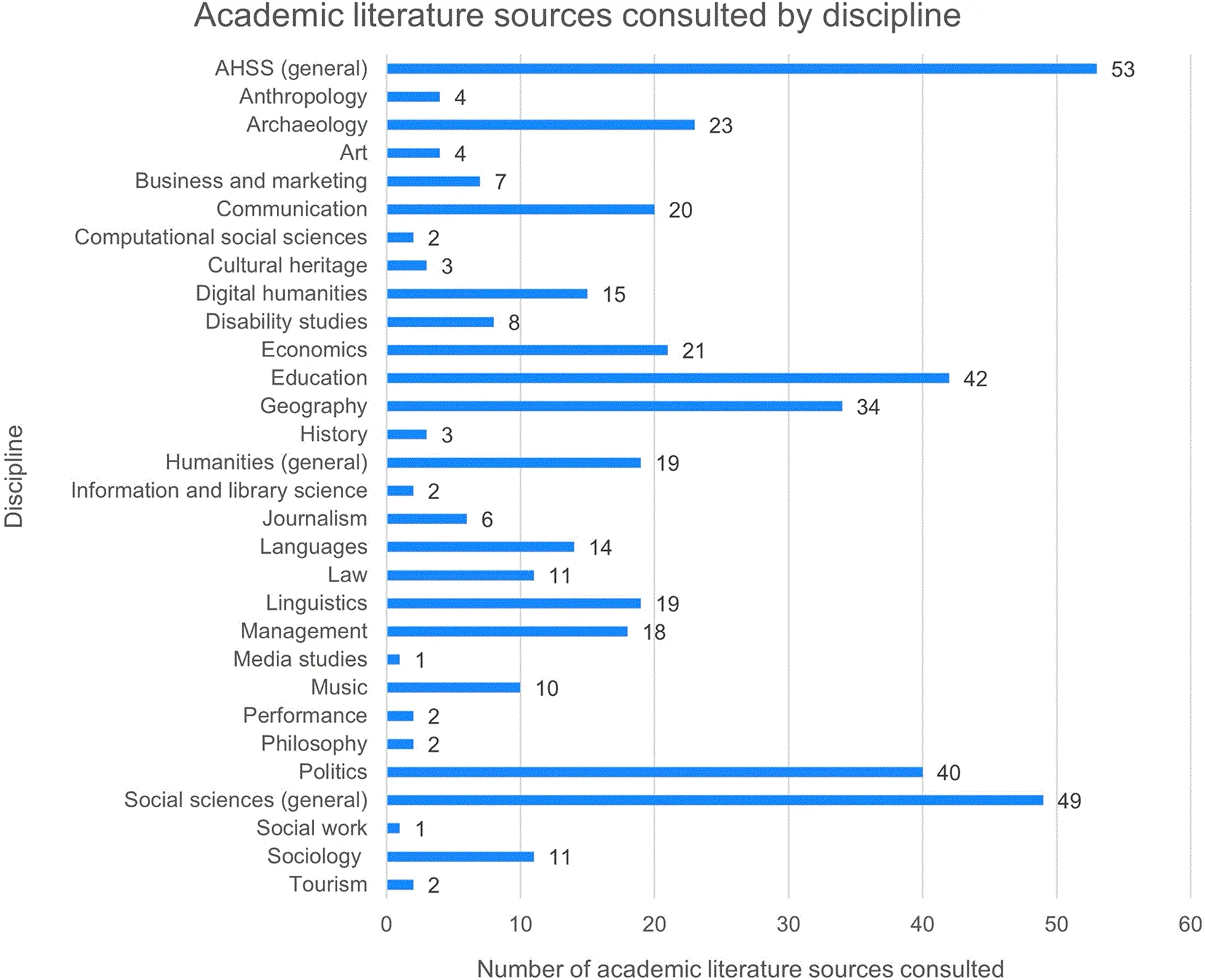
Academic literature sources consulted by discipline. Legend: The figure demonstrates the distribution by discipline of the academic literature sources reviewed as part of the systematic search process for this study. Disciplines listed here are as coded by the research team during the sifting stage of the process.

Results were exported into a shared Zotero library and duplicates merged/removed. During sifting, we reviewed abstracts/titles and classified items using tags as relevant/not relevant according to agreed criteria. Relevant items were coded and tagged by discipline/subdiscipline and practice, where applicable. The full text of items deemed relevant was read and documented in an annotated bibliography. We present a record of the number of items identified through systematic search strategies in Figure 1. A chart detailing the distribution of academic literature items by discipline (as coded during the sifting process) is provided in Figure 2. An additional 51 items were identified via the process of journal scanning.

### Identification and documentation of practices

During the literature review, potential open AHSS practices were recorded together with any examples or additional sources. We identified potential open AHSS practices based on their alignment with the aims of open research in the broadest sense – for example, as articulated in the UNESCO definition – and/or potential additional contexts or senses of openness provisionally identified by the research team. Identified practices were subsequently subjected to targeted research, and where considered relevant, were included in the catalogue. Documentation entailed a short definition of the practice, a full description, openly accessible examples of the practice, and details of relevant resources (e.g. toolkits) and further reading. A second stage then involved conducting a mapping exercise which assumed an inductive thematic approach to identify a typology of openness within which practices may be categorised. The resulting catalogue of open AHSS practices is now available ( MORPHSS, 2026), with the supporting documentation archived in Knowledge Commons ( Adams et al., 2026a). Recognising the limitations of this or perhaps any targeted approach to cataloguing open practices across all AHSS disciplines and research types, the catalogue is open to feedback and suggestions from members of relevant research communities, which will inform future iterations.

## Results and discussion

### Literature review

In this section, we highlight key findings from the literature review, structured according to the research questions addressed, and organised in sections covering the social sciences and the arts and humanities.

### Where, how, and how successfully are existing discourses of ‘open research’ / ‘Open Science’ applied to research practices in AHSS? (RQ1)

***Social sciences***

At both macro (‘the social sciences’) and discipline-specific levels, we found evidence of anxiety about the lack of reproducibility and replicability ^8^ in empirical research in the light of debates in other (particularly STEM) disciplines. At the macro level, examples include Damian et al. (2022), Grossmann (2021), and Balafoutas et al. (2025), the latter of whom identifies a replication crisis in the social sciences that is linked to the proliferation of incentives towards questionable research practices. Anxieties about the lack of reproducibility and replicability were voiced across a majority of individual social science disciplines, with examples including management ( Aguinis et al., 2018; Aguinis and Solarino, 2019; Bamberger, 2019; Bergh et al., 2017; Hensel, 2021) and sociology ( Auspurg and Brüderl, 2022; Breznau, 2021).

These broad concerns are complemented by a body of literature within the majority of social science disciplines calling for the adoption of OS practices in that discipline. In communication, for example, Dienlin et al. (2021) advocate for the adoption of pre-registration, data and code sharing, in addition to a greater frequency of replication studies. A comparable repertoire of practices are suggested by Birkenmaier et al. (2024), Bowman (2024), Lewis (2020), Markowitz et al. (2021), and McEwan et al. (2018). In education, calls to conduct more replications are advanced by Cai (2022) and Makel and Plucker (2014), while adoption of a broader range of OS practices including pre-registration, open data and materials, electronic lab notebooks, and preprinting is recommended in works by Cook et al. (2018, 2021); Gehlbach and Robinson (2018); LeBeau et al. (2021); van der Zee and Reich (2018); and van Dijk et al. (2021). These ‘calls to arms’ are usually voiced by scholars engaged in quantitative research and typically focus on ‘canonical’ OS practices such as pre-registration and registered reports, open data, code and materials, and the conducting of direct replications. Advocacy aimed at researchers is coupled with calls for social science journals to encourage or mandate OS practices to a greater extent, including via adoption of the TOP guidelines (e.g. Alvarez and Heuberger, 2022; Auspurg and Brüderl, 2022; Dadon-Golan and Ziderman, 2024; Dafoe, 2014; Finger et al., 2025; Orozco et al., 2020; Pridemore et al., 2018; Silverstein et al., 2024; Torka et al. 2023).

These examples illustrate a substantial social sciences discourse voicing concerns about a lack of reproducibility and replicability and advocating for the use of canonical OS practices in response. Yet there are also indicators of this agenda’s lack of traction on practice itself. At the macro level, while Ferguson et al. (2023) report increasing uptake of OS practices across social science disciplines based on researchers’ self-reports, Hardwicke et al. (2020) observe only minimal uptake from a random sample of 250 social science articles published between 2014 and 2017, and Jeng and He’s (2022) study of social sciences researchers in US universities found low levels of data sharing. These mixed indications are echoed at a discipline-specific level by findings identifying either low uptake levels or a gap between researchers’ perceptions and use of OS practices – for example, in communication, Haim and Jungblut (2023) and Markowitz et al. (2021); in education, Fleming et al. (2025) and Logan et al. (2025); in geography, Kedron et al. (2024); and in law, Greenspan et al. (2024), Matthews and Rantanen (2025) and Schumann et al. (2019).

***Arts and humanities***

In comparison to the broad application of OS discourses across the majority of social science disciplines, their prevalence in the arts and humanities is both more limited and more localised. Setting aside open access, which is not our key focus, we find more easy alignment with existing OS discourses among subdisciplines with methodological approaches comparable to those in STEM, entailing data-driven, empirical and computational methods. In particular, there have been recent calls for the adoption of Open Science practices such as preprinting, registered reports, and preregistration in applied and corpus linguistics ( Liu, 2023; Liu et al., 2025; Mak, 2024), archaeology ( Queffelec et al., 2023), and experimental philosophy ( Cova et al., 2021).

More generally, arts and humanities researchers often express greater comfort with the term ‘open research’ than ‘Open Science’, especially in Anglophone contexts ( Arthur and Hearn, 2021; Jensenius, 2021). A study by Gualandi et al. (2022) of researchers in disciplines including literary criticism, language and linguistics, the history of art, and archival studies, found mostly positive sentiments towards open research in the humanities, including a ‘willingness to share research data and a desire to “give back” to society the results of research’ (62). We also find some instances of endorsement of the logic of reproducibility and replication as applicable to the humanities in general, most notably in the work of theology and metaresearch scholars Peels and Bouter (2018), including a special issue of the theology journal Zygon dedicated to the topic of replication ( Peels et al., 2024). Issues of reproducibility and replication have also been explored in history ( Huijnen and Huistra, 2022), though these perspectives are not widely adopted within the discipline.

Localised applications of OS discourses include engagement with the potential of open online databases in both media studies ( Taurino, 2022) and art history ( Bylund and Näslund, 2024). Across the disciplines, we found a clear recognition of the need for and benefits of open research data, even where challenges exist in making data open ( Ma, 2024; Gualandi et al., 2022). Linked Open Data (LOD), which allows for targeted aggregation from disparate sources of open data across the web, has furthermore been recognised as ‘one of the most promising directions for the integration of information resources’ in AHSS ( Antopolsky, 2022, 119). Applications of this practice were found in history ( Beretta and Riechert, 2016; Gross et al., 2018; Sturgeon, 2022), fine art ( Buchanan et al., 2011), music ( Rose-Steel and Turnator, 2016), cultural heritage ( Bassett et al., 2022; Kotis et al., 2021), linguistics ( Chiarcos et al., 2011; McCrae et al., 2016), archaeology ( Oksanen et al., 2022), and digital humanities ( Antopolsky, 2022; Brazzo and Rodriguez, 2019; Hyvönen, 2020). This widely-adopted practice is a way of rendering open the dispersed, context-dependent, and specialised data often prevalent in the arts and humanities.

There is limited evidence of engagement in the humanities with the potential of open peer review, as advocated by scholars including Pack Sheffield (2013) in rhetoric and composition and Peters (2014) in philosophy, with particular focus on the opportunities for open participation provided by online platform commenting functions. While Wolfram et al. (2019) found the use of open peer review in humanities journals to be minimal, variants have nevertheless been adopted in some innovative applications in recent years, particularly in the area of experimental publishing ( Adema, 2023; Gallon and Bowman, 2025; Kiesewetter, 2024), with a focus on open peer review as an exercise of care and relationship-building ( Moore, 2025) rather than primarily a transparency practice as is commonly the case in STEM disciplines.

### How and from what perspectives is such an application resisted? (RQ2)

***Social sciences***

We noted above the existence of a significant body of literature advocating the adoption of canonical OS practices in the social sciences to improve transparency, reproducibility and replicability. Yet we also found resistance to this logic and its applicability to all areas of social science research, with particular concerns raised by qualitative researchers, especially those within interpretivist and constructionist traditions. These concerns were found across most disciplines aside from economics, within which qualitative approaches are perhaps less prevalent.

The relevance of replicability and reproducibility to qualitative research is queried at both a macro and discipline-specific level. From an integrative review of discourse on these concepts in qualitative research across disciplines, Cole et al. find that ‘reproducibility [and] replicability, […] are often perceived as in ontological and/or epistemological conflict with qualitative research’ ( 2024, 17). We find this querying in discipline-level debates as well – in geography, for example, Sui and Kedron (2021) question the relevance of reproducibility and replication to ‘ideographic’ work focused on specific cases and contexts. However, there have been some efforts to reframe reproducibility for qualitative research, e.g. in Hanchard and San Roman Pineda’s (2025) concept of ‘re-renderability’, which modifies reproducibility as conditionally defined while still enabling qualitative data to be reused to validate findings. ^9^ The broader underlying principles of OS have also been challenged in such disciplines as communications. Humphreys et al. (2021) highlight the limited relevance of OS principles to interpretive and qualitative research, while Fox et al. note that ‘OS represents certain ontological and epistemological assumptions that marginalize entire areas of research’ (773).

In addition to resisting the logics of reproducibility and replication, we find a related resistance from qualitative perspectives to expectations around specific OS practices, particularly data sharing. ^10^ Resistance to qualitative data sharing is voiced on a variety of grounds: that it will expose participants to risk and may limit participation ( Fox et al., 2021, Monroe, 2018), is epistemically inappropriate in the sense that data in interpretive and big-Q methodologies are not considered separable from the circumstances of their co/creation ( Prosser et al., 2024), entails greater practical challenges than sharing other data types ( Tsai et al., 2016) and requires time and resources that may disqualify disadvantaged researchers from participating ( Dutta et al., 2021; Nordtveit, 2018). These concerns are especially thoroughly addressed in political science, in which qualitative researchers’ resistance to the DA-RT (Data Access and Research Transparency) initiative introduced in the mid-2010s led to extensive reflection in the form of the Qualitative Transparency Deliberations. While working groups on content analysis, text-based sources, comparative methods and process-tracing considered data sharing possible and appropriate to some extent, ethnographers and those working with human subjects and in higher-risk contexts challenged such expectations on the grounds of participant safety and epistemic congruence ( Jacobs et al., 2021). ^11^

We thus identified a considerable body of literature suggesting that the diversity of methodological approaches within the social sciences – with a particular emphasis on qualitative and interpretive approaches – makes it necessary to look beyond the existing OS frameworks when investigating the possibilities of openness in these disciplines.

***Arts and humanities***

As in the social sciences, we find a significant body of literature in the arts and humanities that explicitly or implicitly resists the logic and practices of OS, with scholars highlighting the disparity between STEM-derived OS terminologies and the methods and epistemologies of the arts and humanities. Knöchelmann (2019) and Arthur and Hearn (2021), for example, emphasise the unique characteristics that humanities disciplines share, including their emphasis on perspectivity, subjectivity, historicity and contextual meaning over objectivity and replicability. Ma (2024) highlights the resulting lesser relevance of generalisability in this context, while Kember (2020) foregrounds the contrasting temporalities of STEM research, which benefits from fast dissemination, and those of the humanities, which depend on longer timescales by nature, with implications for the relevance of practices such as preprinting.

These differences have a significant impact on the relevance and applicability of OS practices to arts and humanities research. As in the social sciences, one area highlighted as potentially problematic is open data. Some humanities researchers feel reluctant to use the term ‘data’ at all, perceiving the term as an objectivising one that is embedded in STEM-derived logics ( CO-OPERAS, Bertino and Tóth-Czifra, 2020), while a lack of consensus on what constitutes ‘data’ in these disciplines is elsewhere observed ( CO-OPERAS and Giglia, 2020; Gualandi et al., 2022). The FAIR principles that underpin expectations around data sharing are challenged from arts and humanities perspectives as relying on specific assumptions about the nature of knowledge creation, including the assumptions that data are always digital and always ‘owned’ by researchers ( Tóth-Czifra, 2019). Not only are the materials that inform arts and humanities research not typically understood as or categorised as data ( Hansson and Dahlgren, 2022), common data sources like ‘documents, artifacts, […] newspapers, correspondence, manuscripts, images, and archaeological objects’ often require bespoke and specific curation approaches ( Ma, 2024, 3-4) on brief timescales ( Hansson and Dahlgren, 2022), making sharing challenging.

Further challenges around open data that are highlighted at a macro level include barriers resulting from copyright or restrictive licensing ( Ma, 2024; see also Liem and Mostert (2020) on music research specifically) and ownership of materials by GLAM sector institutions ( Hansson and Dahlgren, 2022). The context-dependency of arts and humanities data ( Ma, 2024; Arthur and Hearn, 2021), like that of qualitative social sciences data, is further highlighted as increasing susceptibility to misunderstanding by future users where data are inadequately documented ( Hansson and Dahlgren, 2022). At a discipline-specific level, highlighted barriers to sharing creative practice research data include ‘issues surrounding informed consent [and] intellectual property’ ( Broadhead and Gonnet, 2025, 32), which can lead to legal and ethical dilemmas, as well as concerns around potential misuse of open data despite the controls enabled by licensing. Jacobs et al. (2024) elsewhere highlight that for practice-based arts researchers, documentation of process is often presumed to be the ‘data’, yet artists themselves may not consider this the research’s most interesting component. In fields such as archaeology, scholars highlight further concerns around political sensitivity ( Fisher et al., 2021) and the potential impact on local communities ( Beale, 2012; Cook, 2018).

Other OS practices such as preprinting, registered reports, and preregistration have received a mixed reception; as regards the former, Laporte (2017) observes low uptake of preprinting in particular. Debates consider what benefits exist here for humanities scholarship and whether humanities scholars would benefit more from rapid formal publication than rapid informal publication via preprints ( Knöchelmann, 2019, 8).

### What existing, emergent and experimental open research practices are taking place in AHSS? (RQ3)

***Social sciences***

From the literature review, we identified two main categories of open research practices in the social sciences that lie outside the conventional scope of OS: those that were explicitly recognised as open practices, and those that were not, but were surfaced by the search terms and exploratory strategies used. The first category includes transparency around positionality (e.g. Schwedler et al., 2019), reflexivity (e.g. Fox et al., 2021; Shaw et al., 2021), member checking or member sharing (e.g. Humphreys et al., 2021), transparency towards participants ( Jacobs et al., 2021), and sharing qualitative research materials ( Renbarger et al., 2023). Authors highlighted these practices to demonstrate the extent to which qualitative and interpretive methods possess existing strategies of openness that are under-articulated in OS frameworks. Practices in the second category included the documentation and dissemination of failure (e.g. Clark and Sousa, 2020) and participatory methods, with a particularly rich seam of material concerning the latter found in geography (e.g. Kinpaisby, 2008; Koopman, 2024; Ritterbusch, 2019) and education (e.g. Ayaya et al., 2020; McGeown et al., 2023, Penuel and Hill, 2019).

***Arts and humanities***

Within the arts and humanities, we noted a distinct focus on forms of openness entailing interaction and collaboration between multiple actors as part of the research process, including in such practices as collective authorship ( Adema, 2021; Hanfling, 2025; Peters et al., 2019) and translation ( Ak and Tekin, 2025; Dickie, 2017; Rosas et al., 2025), as well as experimental modes of longform publishing ( Adema, 2021; Cota, 2023). In addition, innovative means of sharing research processes and research evidence emerge from open documentation of creative methods ( Meece et al., 2017; Messer, 2025), which are often ‘highly complex and multidimensional’ ( Jensenius, 2021), and the open sharing of creative practice outputs ( Bulley and Sahin, 2021; Messer, 2025). Public scholarship is increasingly an important approach towards the accessible communication of arts and humanities research: for example, through academic podcasting ( Beckstead, 2024; Beckstead and Llinares, 2025; Sewell, 2025). Communicating scholarly thought and findings beyond academia ( Arbuckle, 2021) to ‘lay citizen’ audiences is identified as a necessary way for researchers to ‘contribute to the democratization of research’ ( Leavy, 2019), though it could also be argued that accessibility does not in practice straightforwardly equate to democratisation in the sense of a redistribution of epistemic authority. Humanities scholars and interdisciplinary teams are also working creatively with technologies such as Linked Open Data (LOD) to facilitate access to and sharing of cultural and heritage data ( Antopolsky, 2022; Kudera et al., 2024), but also to respond to humanities-specific priorities and enquiries, such as filling the gaps in literary and cultural histories by way of digital enumerative bibliographies ( Coker and Ozment, 2019).

Scholars highlight the communal, processual, experimental ( Adema, 2021; Hope, 2016; Skains, 2018) and heavily context-dependent ( Arthur and Hearn, 2021; Ma, 2024; Knöchelmann, 2019) nature of open research practices in the arts and humanities. On a more practical level, authors also emphasised the need for more robust and sustainable infrastructure for the sharing of nontraditional research outputs (NTROs), often including creative practice works ( Evans et al., 2023; Jackson et al., 2023). What coalesces is the importance of viewing openness in terms of cognition and conception, surfacing new paradigms for developing knowledge ( Stern et al., 2015), and facilitating a more equitable transmission and exchange of ideas within society, beyond the focus on open access and open data ( Arthur and Hearn, 2021).

### Identifying and categorising practices

**Figure 3.**
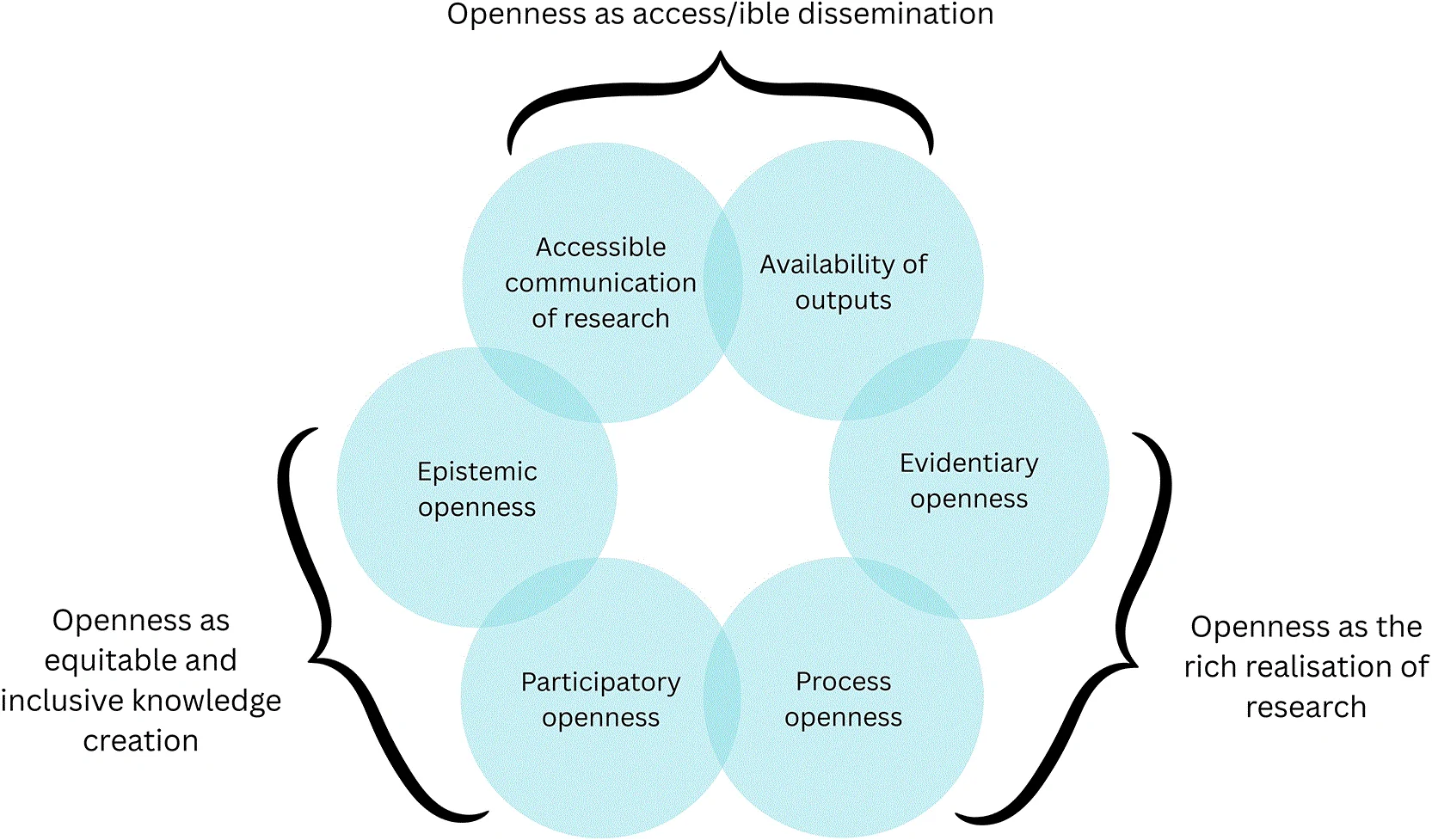
MORPHSS typology of openness in AHSS. Legend: The figure illustrates the six forms of openness that comprise the MORPHSS typology, together with the three broader categories within which these are placed. The presence of overlaps between forms of openness highlights the fact that these may co-occur within a given open research practice, though in reality a wider range of intersections of different forms of openness exists than it is possible to depict here.

**Figure 4.**
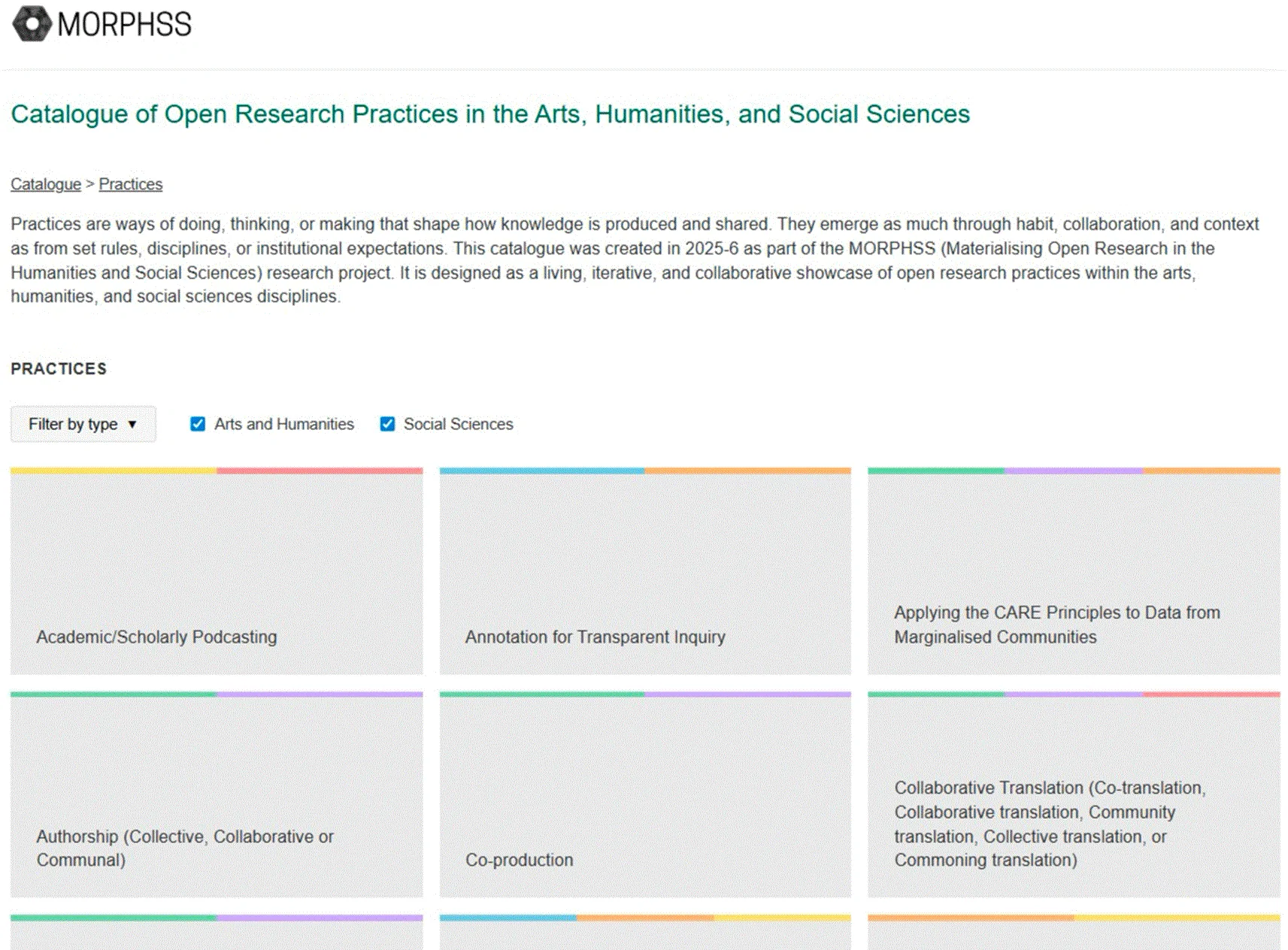
Screenshot from the MORPHSS catalogue (practices page). Legend: The figure illustrates the practices page from the MORPHSS Catalogue of Open Research Practices in the Arts, Humanities and Social Sciences. From this page, users can select individual practices to read more detailed information. Users can also filter by discipline and type of openness.

**Figure 5.**
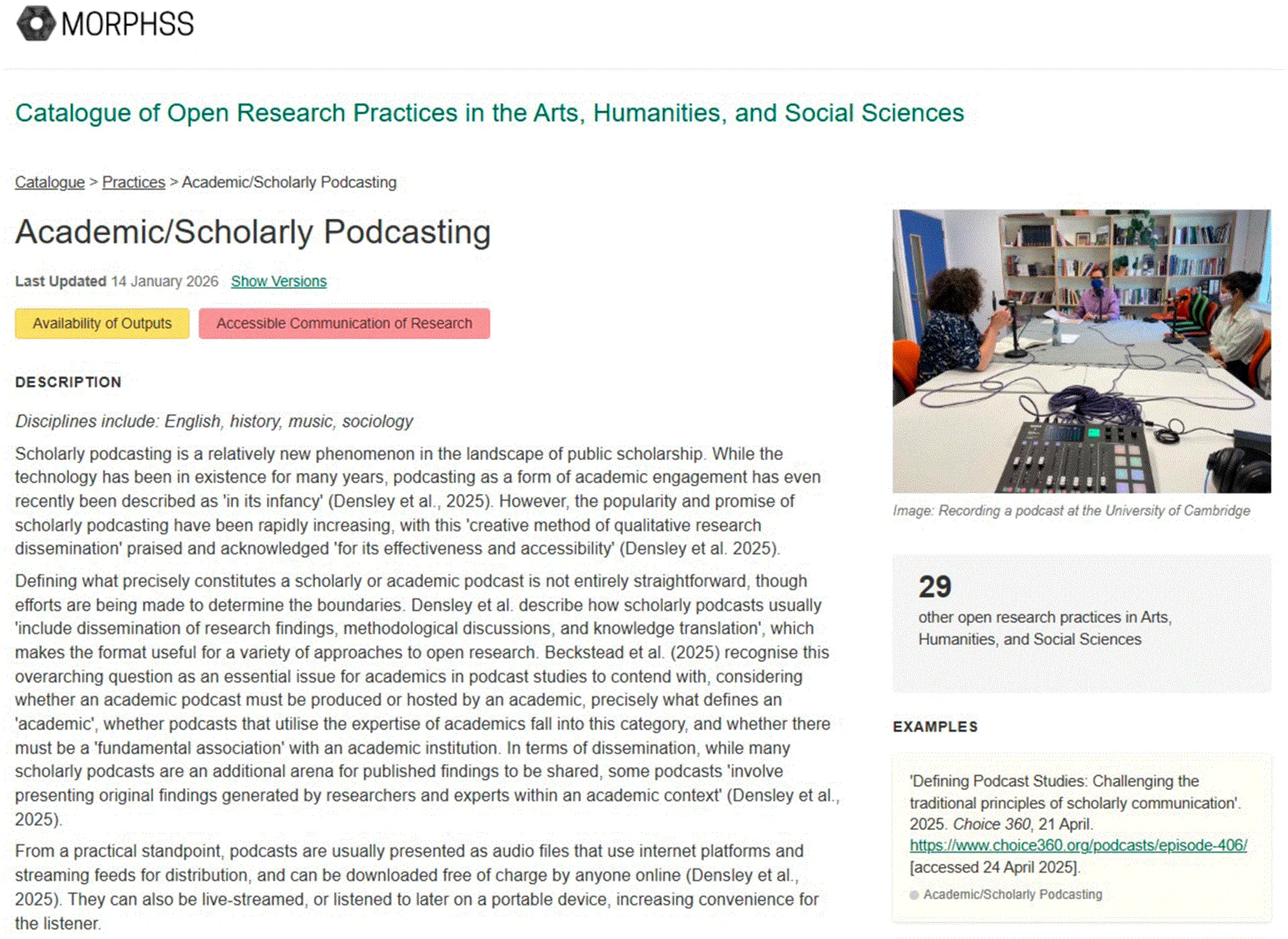
Screenshot from the MORPHSS catalogue (Academic/Scholarly Podcasting). Legend: The figure provides an example of an individual practice page in the MORPHSS Catalogue of Open Research Practices in the Arts, Humanities and Social Sciences. The page includes a detailed description of the practice together with a list of examples and further reading.

The foregoing discussion demonstrated that while the agenda of openness in AHSS has largely been set by and around the OS movement, points of departure exist for an alternative conceptualisation that accommodates the diversity of practice in these disciplines. We here give key examples of these practices in the context of the typology of forms of openness proposed as an outcome of this work. During the mapping exercise, we identified six forms of openness, which we place in three broad categories: Openness as equitable and inclusive knowledge creation; Openness as the rich realisation of research, and Openness as access/ible dissemination. In creating this typology, we drew on vocabularies such as that of the UNESCO Recommendation on Open Science ( 2021) while ensuring their articulation in ways that are inclusive of the variety of knowledge practices occurring in AHSS. An overview of the typology and practices of openness can be found in Figure 3 and Table 2, while a detailed discussion of each practice is provided in the MORPHSS catalogue. We do not reproduce these detailed descriptions here, but provide links to the catalogue for interested readers. Figures 4 and 5 present screenshots from the catalogue to illustrate its format.

**Table 2.** MORPHSS typology of openness in AHSS: overview of practices by type of openness.

		Openness as equitable & inclusive knowledge creation		Openness as the rich realisation of research		Openness as access/ible dissemination	
Open research practice	AH, SS or AHSS	Participatory openness	Epistemic openness	Process openness	Evidentiary openness	Availability of outputs	Accessible communication of research
Academic/Scholarly podcasts	AHSS					•	•
Annotation for Transparent Inquiry (ATI)	AHSS			•	•		
Applying the CARE principles to data from marginalised communities	AHSS	•	•		•		
Authorship (Collective, Collaborative or Communal)	AH	•	•				
Co-production	AHSS	•	•				
Collaborative translation	AHSS	•	•				•
Community-Based Participatory Research (CBPR)	AHSS	•	•				
Data papers	AHSS			•	•	•	
Digital enumerative bibliographies	AH				•	•	
Documenting and disseminating failure	SS			•			
Experimental longform publishing	AHSS	•		•		•	
Linked open data	AHSS				•	•	
Member checking/member sharing	SS	•			•		
Open documentation of creative methods	AH			•	•	•	
Open documentation of methods	SS			•		•	
Open longform scholarship	AHSS					•	
Open materials	AHSS			•		•	
Open peer review	AHSS	•		•		•	
Participatory Action Research	AHSS	•	•				
Positionality (foregrounding of )	SS			•			
Pre-registering qualitative research	SS			•		•	
Preprints and working papers	AHSS					•	
Public scholarship	AHSS					•	•
Reflexivity	SS			•			
Research-Practice Partnerships (RPPs)	SS	•	•				
Sharing creative practice outputs	AH				•	•	
Sharing creatively refigured data	SS				•	•	•
Sharing ethically fabricated data	AHSS				•	•	
Sharing qualitative data	AHSS				•	•	
Virtual exhibitions	AH					•	•

Legend: For each of the open AHSS research practices identified, the table details the forms of openness the practice demonstrates.

It is important to note some key points on the relationship between the practices detailed here and those characteristically identified in STEM disciplines. While some AHSS practices, such as preprinting, open peer review and preregistering qualitative research, have counterparts or equivalents in STEM, in many cases their function and application differ in AHSS contexts. For example, in iterative forms of qualitative research, pre-registration functions primarily to openly disseminate research plans and aid an initial formulation of an approach that will evolve, rather than to curtail later misrepresentations of the research process. These differences and (in some cases superficial) similarities are documented in full in entries in the MORPHSS catalogue, which also place these instances of actual and apparent parity in the context of a broader range of established and experimental open practices in AHSS that do not possess straightforward equivalents in STEM. We also note that the range of open AHSS practices identified is not exhaustive, rather providing a starting point in ongoing efforts to map this terrain.

Below, we further unpack the components of the MORPHSS typology of forms of openness in AHSS.

### Openness as equitable and inclusive knowledge creation

In this category, which correlates to the ‘Open engagement of societal actors’ and ‘Open dialogue with other knowledge systems’ quadrants of the UNESCO Open Science model, we place the following two forms of openness: participatory openness and epistemic openness. We borrow this vocabulary from Pinfield (2024), where it is primarily used to characterise the conditions for equitable open access.

***Participatory openness***

By participatory openness, we refer to practices that open participation in research to a wider group of individuals and communities outside the academy. In practices and approaches such as *Co-production* , *Community-Based Participatory Research* *,* *Participatory Action Research* and *Research-Practice Partnerships* ^12^ in disciplines such as education, sociology, political science and human geography, the involvement of communities and individuals extends beyond merely acting as subjects or passive participants in research ( Aldridge, 2015, 4-5). Rather, participation may and ideally does extend to all aspects of the research process, including the identification of research questions, research design, data creation and analysis, and communication of findings, which can include co-authorship of publications as well as other forms of dissemination such as workshops, blogs and exhibitions ( Bell and Pahl, 2018, 110). Participation in the formulation stages opens research to the concerns of the communities it addresses, increasing its relevance and impact ( Resnik and Kennedy, 2010, 199); involvement in data creation and analysis enables participants’ expert and lived experience knowledges to inform these processes while also providing opportunities for capacity building ( Facer and Enright, 2016, 122-4); and involvement in disseminating research ensures that participants are appropriately credited for their contributions and able to shape forms of dissemination that engage their communities. *Open peer review* in the form of open participation – allowing comments and feedback from broader publics as part of an open evaluation process – offers another instance of participatory openness.

***Epistemic openness***

Epistemic openness designates practices that value and engage with a range of knowledge types and ways of knowing other than those existing solely within the academy. There is a significant overlap here with the practices that embody participatory openness, in that practices that welcome individuals and communities as co-researchers are also often ones that value the forms of knowledge they contribute to research. These forms of knowledge might include lived experience (as in, for example, *Participatory Action Research* , which may engage individuals with experiences of specific forms of marginalisation); communal forms of knowledge including Indigenous knowledges (which may be explored in applications of *Community-Based Participatory Research* ) or professional and practice-based knowledge (e.g. in *Research-Practice Partnerships* ). Methodologies that value and engage these and other forms of knowledge unsettle pre-existing hierarchies of knowing, countering previous extractivist approaches.

Practices of epistemic openness require cultural and epistemic humility ( Ghiso et al., 2019, 11; Ross, 2010) through which researchers acknowledge the contingencies of their own knowledge as well as its embeddedness in exclusionary hierarchies and logics of marginalisation. This can involve processes of ‘unlearning’, ‘co-learning’ and ‘relearning’ ( Dutta, 2019, 2-3), in addition to rigorous reflexive practice ( Groot et al., 2022). Relevant too may be the *application of the CARE principles* for equity in the management and sharing of Indigenous data ( Carroll et al., 2020). The principles – which can also be applied to research with other marginalised communities ( Suchikova and Nazarovets, 2025) – align treatments of Indigenous data with Indigenous peoples’ collective interests and agency, which may entail such practices as co-designing data management processes, using culturally appropriate metadata and ceding control to communities over whether and how their data is shared.

### Openness as the rich realisation of research

This category contains two forms of openness – process openness and evidentiary openness – that denote ways of making available aspects of the research process that have historically been hidden or obscured. We use the language ‘rich realisation of research’ rather than, for example, ‘transparency’, to acknowledge that for some AHSS researchers working in interpretivist and constructionist traditions, ‘transparency’ has instrumental connotations that suggest scrutiny or verification as the primary purpose of sharing; such connotations may be inappropriate where the purpose of research is not framed as the discovery and dissemination of a pre-existing external reality. Our terminology, in contrast, suggests an abundance that manifests the multidimensionality and complexity of research processes, enhancing audiences’ ability to engage with research processes, contexts and materials.

***Process openness***

Process openness refers to sharing elements of the research process, with examples including *open materials* (publishing research materials such as interview guides, participant information sheets, and coding schemes), *open documentation of methods* , *open peer review* , or using a technology like *Annotation for Transparent Inquiry (ATI* *)*, which enables annotation of published articles with detailed notes, to provide further methodological information. *Pre-registering qualitative research* , where it is possible and meaningful to specify approaches in advance, supports discoverability of research plans, while *documenting and disseminating failure* ensures a richer picture of research as it has actually taken place.

We also consider practices like *reflexivity* – continuous self-examination of the impact of (inter) personal context on a study’s design, implementation and analysis – and explorations of *positionality* – clarification regarding the standpoint from which the research is produced – as examples of process openness, insofar as they clarify the conditions under which knowledge was produced. These forms of process openness are well-established in qualitative and AHSS research but less so in quantitative and STEM research, an imbalance scholars including Jamieson et al. (2023) encourage quantitative researchers to address.

***Evidentiary openness***

Evidentiary openness might otherwise have been termed ‘data sharing’, but to do so would be reductive of a diverse set of practices by which research evidence is made available to the communities (peers and participants) of research. Examples include *sharing qualitative data* via a data repository in ways that align with the FAIR principles, which may often require deidentification processes and/or levels of access restriction to be applied, and publishing a *data paper* – a short article documenting a dataset and describing its context, creation, and reuse potential – a practice common to both AHSS and STEM contexts. Evidentiary underpinnings to a published paper can also be highlighted through *Annotation for Transparent Inquiry* . Where identification risks remain or consent is not obtainable, researchers might use more oblique strategies of evidentiary openness including *sharing ethically fabricated data* . Evidentiary openness can also entail *applying the CARE principles* to ensure data and materials are shared appropriately in line with the preferences of marginalised communities, or to ensure such materials are not shared openly but instead returned to communities in a different manifestation of such openness.

Documentation and *outputs from creative practice research* may not be straightforwardly categorisable as ‘data’, but their sharing via mechanisms like archived portfolios likewise achieves evidentiary openness. Finally, *sharing* *creatively refiguring data* – qualitative data refigured using arts-based forms such as drama or poetry – can offer a means of evidentiary openness with participants, as can *member sharing* , in which participants are invited to provide input on the data or analyses produced from their involvement in research.

### Openness as access/ible dissemination

This category brings together two overlapping yet distinct forms of openness that apply to the availability and accessibility of research outputs. Our identification of two forms of openness here highlights the fact that the open sharing of outputs does not necessarily make these outputs accessible to wider publics. Both may be required to realise the benefits of ‘open access’ in the broadest sense.

***Availability of outputs***

In this category, we include open access outputs such as *open longform scholarship* , *data papers* , *open materials* , *pre-registrations* , *open peer review* reports, *preprints and working papers* and *open qualitative data* . In their free and open availability, these enable scholars to access research outputs regardless of their institutional context and its associated level of resource.

***Accessible communication of research***

Accessible communication of research is the dissemination of research in ways that are comprehensible, meaningful and engaging to broader publics, corresponding to the UNESCO pillars of Open Science of ‘open engagement of societal actors’ and ‘open scientific knowledge’. This may include outputs such as *academic podcasts* , freely-available audio recordings that enable research findings to be accessibly presented for diverse audiences. We also include in this category *virtual exhibitions* , freely-accessible digital or online exhibitions, together with other forms of *public scholarship* such as blogs, social media posts, television and radio broadcasts and public lectures. Another means of accessibly communicating research is *sharing creatively refigured data* , for example, in the form of ethnodrama or research poetry.

### Forms of openness and their audiences

**Figure 6.**
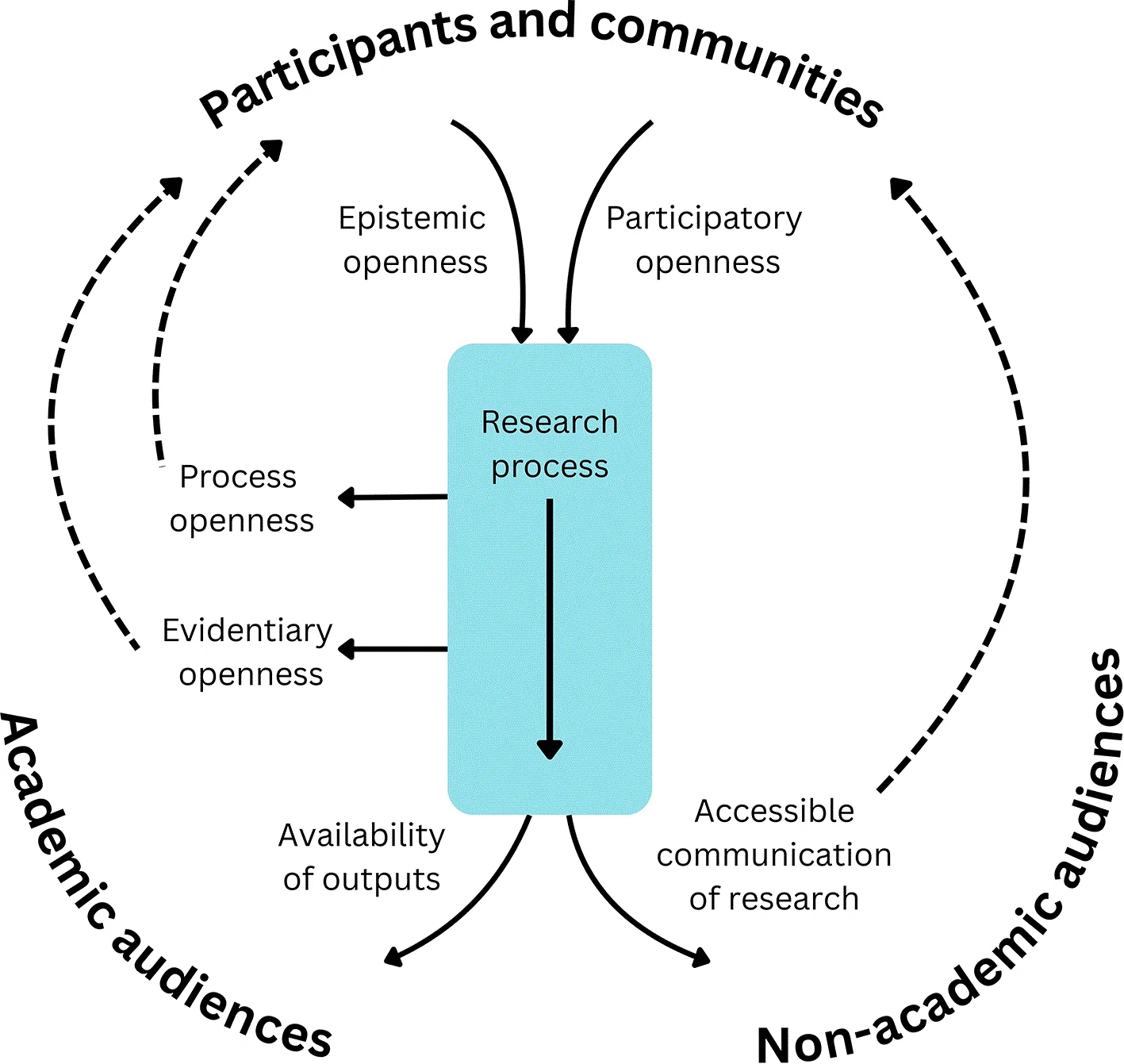
Forms of openness and their audiences, participants and publics. Legend: The figure illustrates the ways in which different forms of openness enable different communities and groups to benefit from and/or contribute to research. Epistemic and participatory openness allow participants and communities to shape and enrich research processes; process openness, evidentiary openness and the availability of outputs primarily benefit academic audiences; and the accessible communication of research primarily benefits non-academic audiences. Dotted lines indicate where additional benefits may occur.

These forms of openness, and the audiences, participants and publics to and with whom they are directed, are summarised in Figure 6:

Practices of epistemic and participatory openness enable participants and communities to input into, and even shape, research processes; these stakeholders may also be the beneficiaries of process openness, evidentiary openness and the accessible communication of research, via practices such as member sharing and public scholarship. Academic audiences benefit largely from practices of process openness, evidentiary openness and availability of outputs, which increase the possibilities for engagement with a study and facilitate future research. Accessible communication of research is the central form of openness to benefit non-academic audiences, via practices such as academic podcasts, virtual exhibitions and other forms of public scholarship.

### Reflections and recommendations

We thus identify six types of openness in AHSS – participatory and epistemic openness, process and evidentiary openness, availability of outputs and the accessible communication of research, a model we refer to as the MORPHSS typology. As noted, this typology in many ways evokes the inclusive model of openness articulated in the UNESCO Recommendation, while theorising its contents and categories in terms that are sensitive to the goals and epistemologies of AHSS research. In this sense, the forms of openness identified here are areas which should also be more fully explored in the context of STEM research – for example, through a greater emphasis on practices of citizen science and science communication and more extensive consideration of the CARE principles. The formulation and application of such a framework to AHSS is especially crucial, however, as the AHSS practices identified here are more broadly and evenly distributed across these different forms of openness than those characteristically highlighted within STEM subjects.

The typology identified here also foregrounds the extent to which, rather than conceiving OR solely as the openness *of* research materials such as data, code and publications, it can also be productively positioned as an openness *to* certain practices in AHSS. For example, many of the forms of openness identified above concern the openness to different participants, knowledges and communities, while other practices such as open peer review and experimental longform publishing emphasise OR’s ability to catalyse new forms of knowledge production. Many AHSS fields have long-established norms around authorship, research practice, and dissemination, shaped by dominant research cultures and assessment mechanisms. Openness, however, as noted elsewhere by Adema (2021), encourages experimentation with these entrenched practices, suggesting the need for research stakeholders to engage with open research in part for its potential to stimulate a plurality of innovative practices in AHSS fields. In this section of the article, we further reflect in brief on some of the implications of this work for an epistemically inclusive approach to open research.

### Open practices in AHSS are diverse and manifold

From a necessarily partial and incomplete survey, we have identified a diverse set of practices, the majority of which lie outside the boundaries of the canonical repertoire of OS. These likewise manifest a much wider set of aims than facilitating reproducibility and replicability, and a wide range of relevant actors, interlocutors and audiences to and with whom openness may be directed. Openness directed towards research participants, and openness directed towards general audiences, are especially emphasised. Regulatory regimes that mandate, measure or encourage open research must take account of the range and variability of open practices across the disciplines, including the fact that:

### Openness in AHSS is not binary and resists quantification

Attempts to automate the monitoring of OS practices via initiatives like the French Open Science Monitor ( Bassinet et al., 2023; Bracco, 2022) and UKRN Open Research Indicators Project ( Jacobs et al., 2026) rely substantially on binary measures (data is shared or not shared, a study has been pre-registered or not pre-registered), and in many instances, on the ability of Text and Data Mining (TDM) to determine whether a given condition has been met. The nature of many open practices in AHSS, however, makes them less conducive to binary and quantitative forms of evaluation. Research is either pre-registered or not, but can be *more or less* participatory, including in superficial ways; there is no quantitative measure of whether the management of data in a project is in line with the CARE principles, and they do not lend themselves to such an approach. There are also several ways in which many of these practices may be achieved, complicating the potential for automated assessment. For example, open methods could take the form of a repository deposit, a methodological appendix, ^13^ or use of a subject-specific interactive digital platform. Potential efforts to regulate AHSS open research are further complicated by a related point:

### The selection and implementation of open practices in AHSS are situated rather than standardisable

A consideration of open AHSS practices such as qualitative data sharing and applications of the CARE principles underscores the degree to which the appropriateness of many of these practices is dependent upon a range of ethical and situational factors, including community preferences and interests. This context-dependency evokes the Open and Collaborative Science in Development Network (OCSDNet) Manifesto’s call for research that ‘practices situated openness by addressing the ways in which *context*, *power* and *inequality* condition scientific research’ ( Albornoz et al., 2017, 294), as well as Sabina Leonelli’s (2023) framing of openness as underpinned by efforts to ‘cultivate epistemic justice as appropriate to the situations of inquiry at hand’ (53, 63). In these framings, open practices are not inherently valuable but only insofar as they facilitate equitable participation in research among the actors and contexts concerned.

This situatedness is evident in the MORPHSS typology in the presence of forms of openness (e.g. participatory and epistemic) that are grounded in ethical relationships and responsibilities. The CARE principles are a writing-large of the fact that recognising and supporting the multiple actors and knowledges at play in research means attuning oneself to contextual specificities and to the agency and interests of others. Practices like participatory methodologies, applications of the CARE principles, member sharing and reflexivity likewise foreground the necessity of a research ethics of care ( Miele et al., 2024) that is at once individually and communally focused. This may at times make practices like data sharing inappropriate, or may instead compel the sharing (returning) of data to participant communities rather than research communities or broader publics. In its turn, this point highlights the fact that in AHSS there are echelons or realms of potential openness rather than a single appropriate level and direction of openness (i.e. unrestricted openness to ‘all’, for a value of ‘all’ which presupposes a level of empowerment – social, linguistic, educational, technological – necessary to experience its benefits). Implications of this context-specificity include the fact that, as Sarahanne Field argues with respect to qualitative research, open research ‘must reject blanket mandates of sharing in favour of situated, reflexive evaluation’ ( 2025, 22-23).

The appropriateness of particular open AHSS practices is also often specific to the methodological approach and (sub)discipline concerned: for example, not all AHSS projects involve collecting or creating primary data or working with participants, making data sharing and participatory methods less relevant in some contexts. Selection is dependent, too, on questions of methodological congruence; as Steltenpohl et al. (2025, 1) note, ‘[e]ngagement or disengagement of any given practice should be guided by demands of research epistemology, context, community, topic and methodology’. As discussed above, a practice like pre-registration may be less meaningful or productive in the context of iterative and co-creative research methodologies which resist pre-specification, while notions of data sharing are considered by many qualitative researchers to presuppose too much in terms of data’s portability and the meaningfulness of attempts to de- and re-contextualise them. A consideration of openness that is genuinely attuned to the diversities of practice is compelled to acknowledge that as Tamminen et al. state, the appropriateness of a given open practice ‘must be considered in the context of each study and the evolving relationships among researchers, participants, and communities’ ( 2021, 871), with researchers retaining agency in this process.

### Recommendations

Considering the open AHSS practices identified in this project, we note that openness in AHSS is manifold, often irreducible to binaries or quantitative measurement, and frequently situated rather than standardisable, making flexibility a necessary part of efforts to encourage and evaluate it. These reflections prompt a set of initial recommendations for key stakeholders in the following categories: funders, open research monitoring initiatives, institutions, publishers, learned societies, and researchers; we list these in full in the Appendix with some key points highlighted below.

For stakeholders in the regulatory regimes of open research – those mechanisms by which openness is encouraged, mandated or measured, which include funder and journal policy, institutional hiring, reward and recognition processes, and open research monitoring initiatives, we recommend reviewing the scope of open practices currently stipulated, assessing their level of inclusiveness across disciplinary, epistemological, and methodological differences. Where current policies and processes lack inclusivity, processes of revision should involve AHSS communities and include the development of exemplars to guide appropriate practice. Crucially, we recommend the need for flexibility in revised regulatory mechanisms: for example, funding schemes might refer applicants to comprehensive information on a wide range of OR practices and require a statement detailing which will be used in the proposed study and why, evaluating this component on the basis of congruence between the practices selected and the project’s methodological approach and epistemological stance. ^14^ Journal policies, especially in AHSS and interdisciplinary areas (e.g. sustainability, development), might likewise provide an overview of a broad and inclusive range of OR practices and require authors to provide an OR statement on article submission that details which of these practices they have employed and why, with links to evidence. In this, we follow Steltenpohl et al.’s (2023, n.p.) suggestion that ‘[b]y expanding open science guidelines to leverage a broader array of rigor and transparency-promoting practices (e.g., reflexivity), we can truly begin to advance practices.’ Finally, we acknowledge the implications of this work for open research monitoring and evaluation, foregrounding tensions between existing institutional reliance on standardisation and quantification and the recognition of diverse, context-appropriate open practice. We suggest that greater alignment of approaches to open research monitoring with the UNESCO-affiliated OSMI principles ( Bobrov et al., 2025) – which emphasise the need for indicators to be co-created with research communities, inclusive of diverse knowledge systems and epistemologies, and adaptable to specific research contexts – would be beneficial.

For AHSS researchers, we emphasise the importance of actively exploring a broad range of open practices beyond those expressly mandated, selecting those that are consistent with epistemological and methodological approaches and appropriate to research contexts, and actively contributing to debate regarding appropriate forms of openness in AHSS. We also encourage researchers to evaluate their current practice through the lens of openness, and to advocate for the recognition of the openness of aspects of their work that may be unrecognised within current frameworks.

## Conclusions and limitations

The scope and comprehensiveness of the MORPHSS catalogue is limited by the capacities of the small research team within the project’s time constraints, resulting in a necessarily partial mapping of these practices. Using a literature review approach as the basis for such efforts risks uncovering only those aspects of practice that are already framed in a vocabulary of openness; our use of exploratory strategies sought to mitigate this but may not have wholly redressed the balance between the recognised and the as-yet-unrecognised. The framing of the project in terms of discipline rather than research type also carries potential limitations in grouping together what is actually a diverse range of methodologies and approaches; we have clarified the reasons for this choice. Despite these limitations, we feel this work offers a valuable starting point in efforts to reimagine open research for the arts, humanities and qualitative social sciences, in particular by highlighting the multiplicity of practices that lie outside current dominant models of Open Science. Emphasising this diversity carries the potential to enrich debates around open research at a multidisciplinary level, problematising and diversifying existing models, promoting epistemic and disciplinary pluralism, and inviting reconsideration of approaches to openness across all areas, including STEM fields, which might further consider the relevance of some of the broader forms – and goals – of openness highlighted here.

## Pre-registration statement

This research was not pre-registered.

## Rights retention statement

For the purpose of open access, the authors have applied a Creative Commons Attribution (CC BY) licence to any Author Accepted Manuscript version arising from this submission.

## AI statement

No AI tools have been used at any stage in the writing of this article or in the underlying research.

## Data Availability

The following data and methodological materials are openly available: Knowledge Commons: ‘Data for the MORPHSS Catalogue of Open Research Practices in the Arts, Humanities and Social Sciences’. https://doi.org/10.17613/pt306-c8c15 ( Adams et al., 2026a). This contains the following underlying data:
• Full descriptions - AHSS open practices (MORPHSS catalogue) - text for the full descriptions of AHSS practices included in the MORPHSS catalogue ( MORPHSS, 2026) (available in both .docx and .txt formats) • MORPHSS catalogue content AHSS open practices - tabulated data containing details of each practice included in the MORPHSS catalogue ( MORPHSS, 2026), comprising short definitions, disciplines, types of openness, examples and resources/further reading (available in both .xslx and .csv formats) Data are available under the terms of the CC-BY licence Creative Commons Attribution 4.0 International (CC-BY-4.0) license. Knowledge Commons: MORPHSS WP 1 Literature review supporting materials. https://doi.org/10.17613/475rc-c0j16 ( Adams et al., 2026b). This contains the following methodological materials:
• Literature review protocol (protocol for the literature searches underlying the research presented in this article) (available in both .pdf and .txt formats) • Bibliography of academic literature consulted as a result of systematic searches (available in both .xslx and .csv formats) Methodological materials are available under the terms of the CC-BY licence Creative Commons Attribution 4.0 International (CC-BY-4.0) license. The Appendix can be found here: Adams, Jenni, Miranda Barnes, Samuel Moore, and Stephen Pinfield. 2026. ‘Appendix to Cataloguing and Theorising Open Research Practices in the Arts, Humanities and Social Sciences: Problematising and Diversifying 'Open Science' – Recommendations’. Knowledge Commons. https://doi.org/110.17613/gchaj-4tq29 ( Adams et al., 2026c) Data are available under the terms of the CC-BY licence Creative Commons Attribution 4.0 International (CC-BY-4.0) license.

## Footnotes

^1^
While the term ‘Open Science’ is often used interchangeably with ‘open research’, we use the latter in this article to emphasise a distinction between the broader phenomenon of open research and STEM-focused conceptions of openness.

^2^
See also de Oliveira et al. (2021, 787) and Rafols et al. (2024, 4).

^3^

Grant et al. (2025, 3) note that the revised 2025 TOP ‘includes a conceptual framework [...] to clarify its primary objective — promoting the verifiability of empirical research claims’, suggesting that in fact, TOP is aimed at specific contexts and goals within open research rather than universal applicability. Nevertheless, as these authors note, the original ‘TOP 2015 lacked an explicit conceptual framework’, arguably (alongside the general terminology of ‘Transparency and Openness’ in the name) rendering it susceptible to precisely such problematic universalisation.

^4^
This source was selected for its provision of a granular and comprehensive list of AHSS disciplines.

^5^
E.g. Bennett (2021), Huma and Joyce (2023), Pownall et al. (2023).

^6^
While open access to journal articles, being already well-documented, is not a key area of focus in the project, open longform publishing is included in the catalogue as an emergent practice; material on this practice was gathered through targeted searches.

^7^
These were *Accountability in Research*, *IASSIST Quarterly*, *International Journal of Qualitative Methods*, *International Journal of Social Research Methodology*, *Journal of Electronic Publishing*, *Publications (Basel)*, *Qualitative & Multi-method Research*, *Qualitative Inquiry* and *Qualitative Research: QR.*

^8^
While the definition of these concepts varies, we follow the usage that ‘[r]eproducibility is obtaining results consistent with a prior study’ by using the original data and analytic techniques, while replication occurs when ‘new data are collected and the same, or similar, methods are used to produce a [comparable] result’ ( Sui and Kedron, 2021, 1276). Both concepts depend on sufficient transparency of data and methods in the original study, and both presuppose the research’s independence from the researcher’s standpoint.

^9^
On this point, see also Cole et al. (2024, 16).

^10^
Concerns were also raised about the sharability of specific data types such as proprietary data ( Hensel, 2021) social media data ( Bishop and Gray, 2017), and image data ( Late et al., 2024).

^11^
For a series of individual dissenting perspectives in political science, see also Cramer (2018), Hirschmann (2018), Majic (2018), and Tripp (2018).

^12^
These are only a small selection of a much wider group of participatory research practices, which it was not possible to catalogue within the constraints of the project.

^13^
Though the former is preferable from a discoverability and preservation point of view, there is a long history of such appendices in publications like ethnographic books.

^14^
In our framing of this point in terms of congruence, we draw on Braun and Clarke’s (2025, 414-5) emphasis on reflexive openness regarding methodological congruence as a point of focus in evaluating research.
