# Supplementary material for: Cataloguing and Theorising Open Research Practices in the Arts, Humanities and Social Sciences: Problematising and Diversifying ‘Open Science’
Source: F1000Res. 2026 Aug 20;15:515. Originally published 2026 Apr 15. [Version 2] doi: 10.12688/f1000research.178353.2 (PMC13287985; doi:10.12688/f1000research.178353.2)
Supplement: Peer review history

# Reviewer report: Reviewer response for version 2

**DOI:** 10.5256/f1000research.206863.r512911

**Published:** 2026-08-21

**Authors:** Simon Dumas Primbault

**Affiliations:** 1 OpenEdition Lab (CNRS), Marseille, France

I have no further comment. Thank you for having taken the time to critically engage with the evaluations, and thank you for this work!

Is the work clearly and accurately presented and does it cite the current literature?

Yes

If applicable, is the statistical analysis and its interpretation appropriate?

Not applicable

Are all the source data underlying the results available to ensure full reproducibility?

Partly

Is the study design appropriate and is the work technically sound?

Partly

Are the conclusions drawn adequately supported by the results?

Partly

Are sufficient details of methods and analysis provided to allow replication by others?

Partly

Reviewer Expertise:

History and sociology of research practices in the humanities and social sciences, more specifically in an open science regime. Currently working specifically on open research data practices in HSS.

I confirm that I have read this submission and believe that I have an appropriate level of expertise to confirm that it is of an acceptable scientific standard.

# Reviewer report: Reviewer response for version 1

**DOI:** 10.5256/f1000research.196728.r487827

**Published:** 2026-06-22

**Authors:** David Mellor

**Affiliations:** 1 Center for Open Science, Charlottesville, Virginia, USA

In "Cataloguing and theorising open research practices in the arts, humanities and social sciences: Problematising and diversifying ‘Open Science’" the authors provide a comprehensive review of open research practices in the arts and humanities. The work provides a substantive discussion on the limits of many existing recommendations in the open science movement, documents responses to them from outside of STEM communities,  and provides recommendations to policy makers. Below are some minor comments that I hope will be helpful to the authors.

 In their comments about existing open science policies, the authors discuss TOP: "Such universalisation of expectations is also evident in Open Science guidance and advocacy materials such as the apparently discipline-neutral TOP guidelines, which aim to inform journal policy on openness yet focus primarily on transparency around materials, data and code.” TOP provides recommendations for policy makers who wish to increase the verifiability of empirical research claims (see suggested citation - Ref 1 - Grant S, et al., 2025). This specific motivation and purpose leads to some of the irrelevancy of TOP to disciplines where those are not specific concerns. 

 On a related note, I recommend that the authors provide readers with guidance on how to select areas from MORPHSS based on the motivating concern of the policy maker. As has been widely discussed, TOP (as a primary example from the STEM disciplines) comes from concerns about replicability (being able to conduct a study again and come to a similar conclusion), and reproducibility (coming to the same conclusion from the same data that the first author used), and thus recommends practices that increase verifiability. This is one component of overall credibility and trust, but is not comprehensive; for example, calls for increasing transparency into peer review may increase trust in research (by seeing how much or little review occurred prior to indexing) even if it does nothing to increase the verifiability of research claims. This distinction is important if the policy maker wants to address a specific perceived shortcoming, and I believe provides helpful context for them in developing policies. As such, a helpful addition to the authors' recommendations would be to outline various problems in arts and humanities research caused by opaque research practices and to match those concerns with specific recommendations from the MORPHSS list of options. To my knowledge there is not a corresponding lack of reproducibility or replicability in the arts and humanities, which is one reason why calls to action in STEM do not resonate with methods or processes in other disciplines. 

 The authors assert that this work was not pre-registered, but they did note that their search strategy was published in the knowledge commons ahead of time. The protocol is fairly well detailed and specific to this project, I recommend the authors point to it in their open practice disclosure: https://works.hcommons.org/records/1q09z-e5g62

Is the work clearly and accurately presented and does it cite the current literature?

Yes

If applicable, is the statistical analysis and its interpretation appropriate?

Yes

Are all the source data underlying the results available to ensure full reproducibility?

Yes

Is the study design appropriate and is the work technically sound?

Yes

Are the conclusions drawn adequately supported by the results?

Yes

Are sufficient details of methods and analysis provided to allow replication by others?

Yes

Reviewer Expertise:

Ecology, citizen science (public participation in research), open science, meta-science

I confirm that I have read this submission and believe that I have an appropriate level of expertise to confirm that it is of an acceptable scientific standard.

## References

[1] : TOP 2025: An Update to the Transparency and Openness Promotion Guidelines.2025; 10.31222/osf.io/nmfs6_v2 10.31222/osf.io/nmfs6_v2 PMC1343976442557573

# Response

**Published:** 2026-08-07

**Authors:** Jenni Adams

**Affiliations:** The University of Sheffield Information School, Sheffield, England, UK

*In their comments about existing open science policies, the authors discuss TOP: "Such universalisation of expectations is also evident in Open Science guidance and advocacy materials such as the apparently discipline-neutral TOP guidelines, which aim to inform journal policy on openness yet focus primarily on transparency around materials, data and code.” TOP provides recommendations for policy makers who wish to increase the verifiability of empirical research claims (see suggested citation - Ref 1 - Grant S, et al., 2025). This specific motivation and purpose leads to some of the irrelevancy of TOP to disciplines where those are not specific concerns.*

 We agree that the universalisation of ‘openness standards’ that are in fact limited in their applicability to particular goals and contexts is problematic. However, we view such universalisation as an attribute of dominant discourses around open science and therefore a legitimate point of critique. In other words, although it may be the case that the goals of the TOP principles are to focus solely on practices in support of verifiability of empirical research claims and to be open about this specific scope, we suggest that this has not prevented the principles from being perceived as part of the dominant discourse around openness and subsequently as exemplars for openness in its totality, even in contexts where this is inappropriate. The source referred to here (Grant et al. 2025) also acknowledges that while ‘TOP 2025 includes a conceptual framework [...] to clarify its primary objective — promoting the verifiability of empirical research claims’, ‘TOP 2015 lacked an explicit conceptual framework, which might have led to different interpretations of its goal and scope’ (p. 3). This might be interpreted, in part at least, as a nod to the susceptibility of the framework to universalising deployments in which this limited scope of open practices stands in for ‘transparency and openness’ per se - a usage also arguably invited by the initiative’s name. We have added an additional footnote in the article to clarify our stance and accommodate some of these nuances.

*On a related note, I recommend that the authors provide readers with guidance on how to select areas from MORPHSS based on the motivating concern of the policy maker. As has been widely discussed, TOP (as a primary example from the STEM disciplines) comes from concerns about replicability (being able to conduct a study again and come to a similar conclusion), and reproducibility (coming to the same conclusion from the same data that the first author used), and thus recommends practices that increase verifiability. This is one component of overall credibility and trust, but is not comprehensive; for example, calls for increasing transparency into peer review may increase trust in research (by seeing how much or little review occurred prior to indexing) even if it does nothing to increase the verifiability of research claims. This distinction is important if the policy maker wants to address a specific perceived shortcoming, and I believe provides helpful context for them in developing policies. As such, a helpful addition to the authors' recommendations would be to outline various problems in arts and humanities research caused by opaque research practices and to match those concerns with specific recommendations from the MORPHSS list of options. To my knowledge there is not a corresponding lack of reproducibility or replicability in the arts and humanities, which is one reason why calls to action in STEM do not resonate with methods or processes in other disciplines.*

 The scope and aims of the article are to explore and document where open practices are being used in AHSS subjects, rather than seeking to match existing practice with problems that may be perceived by policymakers; doing so would entail substantial additional work. As a result, we feel this suggestion is outside the scope of this piece of work - though we are considering ways to engage with policymakers elsewhere during the course of the MORPHSS project. An additional (and to the current piece of work, also peripheral) question would be the extent to which approaches to openness are, or are not, by necessity ‘problem-oriented’ rather than conceived in terms of a pursuit of broader benefits or facilitation of more inclusive research communities - i.e. oriented towards abundance rather than the prevention of questionable practice. While to some extent this is merely a question of framing, the MORPHSS project has largely sought to take an approach that looks beyond solely problem-oriented considerations of openness.

*The authors assert that this work was not pre-registered, but they did note that their search strategy was published in the knowledge commons ahead of time. The protocol is fairly well detailed and specific to this project, I recommend the authors point to it in their open practice disclosure: https://works.hcommons.org/records/1q09z-e5g62*

 We would like to clarify that while the literature review protocol was created in advance of the searches being conducted, it was not openly deposited until the article itself was in preparation - in other words, we consider this an open material contextualising the research process rather than a time-stamped pre-registration. We believe that it is appropriately categorised as such in the open disclosure sections as things stand.

# Reviewer report: Reviewer response for version 1

**DOI:** 10.5256/f1000research.196728.r487822

**Published:** 2026-06-15

**Authors:** Simon Dumas Primbault

**Affiliations:** 1 OpenEdition Lab (CNRS), Marseille, France

This article aims to document in detail the inadequacies between the dominant "open science" regulatory frameworks inherited from STEM, and the diversity of actual "open research" practices in the AHSS. It relies on the comprehensive and qualitative review of more than 400 publications on the subject, assembled through keyword searches in various databases (incl. grey literature) and manual exploratory searches (citation tracking, snowball effect...). From this extensive literature review, the authors aim at i) highlighting the frictions between institutional regulatory framework for open science and actual open research practices in AHSS; and ii) draw a catalogue of open research practices specific to the AHSS that are currently undervalued or even put at risk by open science policies.

 Such an endeavour is of great importance for the future of open science policies and infrastructure, as well as for the survival and development of AHSS qualitative practices that are indeed under threat of being discarded both by the current open science regulatory frameworks but also, more broadly, by a general push towards ever more quantitative and computational approaches (usually deemed more scientific). In this regard, this contribution succeeds with brio in, first, meaningfully synthesising the criticisms and resistances raised by a wide range of AHSS scholars against the dominant norms of open science (while nonetheless agreeing in principle with openness) and, second, in displaying the diversity and complexity of a set of open research practices in AHSS through a substantive catalogue available on the website of the project (N.B.: this catalogue would benefit from a more thorough presentation in the article to appeal to reader, for example with a box containing one of its entries as an example). Thus, the article reopens the meaning of openness beyond dominant acceptations, giving flesh to UNESCO's more inclusive understanding of it, and, more specifically, in pointing the fundamentally socio-epistemic nature of open research practices (too often reduced to mere technicalities that could be solved with infrastructure and legal frameworks). Among the merits of this article is its way of clarifying research processes in AHSS and related open research practices, while not reducing their complexity and diversity, which, beyond very specific and detailed case studies, provides a discourse that can be concretely actionable and, indeed, used to produce recommendations that can be found in detail in the project's report. An example of this approach can be found in the authors' rephrasing of some formulas of open science (e.g. "data sharing" to "evidentiary openness"; or "transparency" to "rich realisation of process") thereby recovering the wealth and diversity of AHSS open research practices.

 Here are some comments to further strengthen this important contribution:

 1) About "data"

 - The corpus of literature could be further described, notably a histogram with the publication dates, a pie chart with disciplines and subdisciplines, another with the nature of the publication (peer-reviewed article, research report, institutional report, grey literature, etc.), maybe some information about the status of the authors (in a very recent study we led in my group on open research data in the AHSS, we realised that senior researchers could be more vocal about its inadequacies, or that criticisms were more salient in political sciences than overall), maybe some information about the genre (essay, empirical study, op-ed, conceptual analysis...)

 - Consequently, the full annotated bibliography could be shared as part of the dataset in a csv format.

 - The files shared in the appendix should also be shared in open formats such as csv for the spreadsheet and txt (possibly in markdown) for the catalogue.

 2) About methodology

 - The mapping exercise that was deployed to produce the typology is not described. I reckon it was a rather inductive and collective process that it is difficult to objectify but some more detail about how the categories emerged, possible ambiguities, further information about how the UNESCO guidelines were used, would strengthen the methodology part.

 - The flow diagram of figure 1 could be included in the supporting document on literature review.

 - About the catalogue, it seems to contain objects of different natures: some are material outputs, others are actual practices, others again are more akin to epistemic virtues (such as reflexivity or positionality which, one could argue, are a the heart of every scientific endeavour either AHSS or STEM); so as a reader I wonder if this is deliberate (and therefore would benefit from a comment) or not (and it would be interesting to see in what ways everything could be rephrase as practices using gerunds and what that means for the attached material outputs or epistemic virtues, as is the case for "positionality (foregrounding of)").

 - Maybe a more serious methodological comment is that it is unclear to me how the same corpus can be used for both lines of research: i) synthesising criticisms of open science regulatory frameworks; and ii) cataloguing open research practices. While the first line is quite clear to me as it seems that most of the corpus is composed of reflexive and critical literature produced about open science in the AHSS, I would have thought the second line of inquiry would be addressed through ethnographic methods such as interviews or observations. Which begs the question: what is the link between the practices identified and the texts in the corpus? Are they the practices actually deployed by the authors of the articles in the context of their research? Or are they merely mentioned in the text? If so, in depth or in passing? And consequently, how can they be mobilised in a relevant manner to draw such a catalogue? Overall, this comment raises the issues of corpus building (e.g. why not be interested in all research practices in AHSS and only then discriminating which can be deemed open, rather than relying on declarations of openness?) and the limits of corpus analysis (e.g. how to extract actual practices from a non-dedicated corpus?) which are both addressed in the conclusion but possibly in too little details.

 3) Lastly, but beyond the scope of this article, I wonder about the STEM origins of dominant open science regulatory frameworks: although I agree with the authors that these frameworks are focused on narrowly quantitative and positivist epistemologies, do these really come from STEM? Or rather from institutional actors who have a very simple understanding of research practices in STEM reduced to formal procedures, and consequently write policies or design infrastructure on these premises? Works from Leonelli, for example, show how notions of replicability are heatedly debated even within some subdisciplines of the STEM, which would argue for the fact that AHSS are not fundamentally different and, rather, highlight fundamental epistemic issues that also feature in STEM (although less saliently due to the fact that they were historically more formalised over the past two centuries). Beyond the scope of this article, a more detailed sociological analysis of the circulation of such an abstract epistemology, and of the actors circulating it, would be of great interest.

Is the work clearly and accurately presented and does it cite the current literature?

Yes

If applicable, is the statistical analysis and its interpretation appropriate?

Not applicable

Are all the source data underlying the results available to ensure full reproducibility?

Partly

Is the study design appropriate and is the work technically sound?

Partly

Are the conclusions drawn adequately supported by the results?

Partly

Are sufficient details of methods and analysis provided to allow replication by others?

Partly

Reviewer Expertise:

History and sociology of research practices in the humanities and social sciences, more specifically in an open science regime. Currently working specifically on open research data practices in HSS.

I confirm that I have read this submission and believe that I have an appropriate level of expertise to confirm that it is of an acceptable scientific standard, however I have significant reservations, as outlined above.

# Response

**Published:** 2026-08-07

**Authors:** Jenni Adams

**Affiliations:** The University of Sheffield Information School, Sheffield, England, UK

*The catalogue would benefit from a more thorough presentation in the article to appeal to reader, for example with a box containing one of its entries as an example*

 We appreciate this suggestion. We have added two screenshots from the catalogue as additional figures in the article.

*About "data" - The corpus of literature could be further described, notably a histogram with the publication dates, a pie chart with disciplines and subdisciplines, another with the nature of the publication (peer-reviewed article, research report, institutional report, grey literature, etc.), maybe some information about the status of the authors (in a very recent study we led in my group on open research data in the AHSS, we realised that senior researchers could be more vocal about its inadequacies, or that criticisms were more salient in political sciences than overall), maybe some information about the genre (essay, empirical study, op-ed, conceptual analysis...)*

 We would like to clarify that the exercise conducted was a literature review rather than a Systematic Review, for which the suggested granularity of coding and reporting would be most appropriate. However, we have added a figure to the article showing the distribution of the literature by discipline in order to add important context to the results reported. We appreciate the suggestion and feel this adds a useful dimension to the reporting of the literature review.

*Consequently, the full annotated bibliography could be shared as part of the dataset in a csv format.*

 We have shared the bibliography in unannotated form as part of the literature review protocol deposit (now renamed ‘MORPHSS WP1 Literature Review supporting materials’). Annotations were not produced for intended sharing and would require substantial preparation in order to be rendered meaningful for a broader audience.

*The files shared in the appendix should also be shared in open formats such as csv for the spreadsheet and txt (possibly in markdown) for the catalogue.*

 We have added versions using these file formats to the existing data deposit.

*About methodology - The mapping exercise that was deployed to produce the typology is not described. I reckon it was a rather inductive and collective process that it is difficult to objectify but some more detail about how the categories emerged, possible ambiguities, further information about how the UNESCO guidelines were used, would strengthen the methodology part.*

 Indeed, this is an accurate characterisation of the process - we have made an addition to the wording used to describe it in order to highlight these aspects.

*About the catalogue, it seems to contain objects of different natures: some are material outputs, others are actual practices, others again are more akin to epistemic virtues (such as reflexivity or positionality which, one could argue, are a the heart of every scientific endeavour either AHSS or STEM); so as a reader I wonder if this is deliberate (and therefore would benefit from a comment) or not (and it would be interesting to see in what ways everything could be rephrase as practices using gerunds and what that means for the attached material outputs or epistemic virtues, as is the case for "positionality (foregrounding of)").*

 We have taken an inclusive approach to the identification of open practices for AHSS that emphasises processes and outputs of varying levels of specificity. We would counter the suggestion that positionality and reflexivity are epistemic virtues rather than tangible components of the research process; these take a range of forms in qualitative research such as specific forms of reflective writing (memo-ing, journalling etc, positionality statements, and collaborative reflection, as highlighted in the catalogue. When documented or otherwise foregrounded in presentations of the research, we suggest, these become open research practices comparable to other, more familiar practices focused on transparency regarding the research process.

*Maybe a more serious methodological comment is that it is unclear to me how the same corpus can be used for both lines of research: i) synthesising criticisms of open science regulatory frameworks; and ii) cataloguing open research practices. While the first line is quite clear to me as it seems that most of the corpus is composed of reflexive and critical literature produced about open science in the AHSS, I would have thought the second line of inquiry would be addressed through ethnographic methods such as interviews or observations. Which begs the question: what is the link between the practices identified and the texts in the corpus? Are they the practices actually deployed by the authors of the articles in the context of their research? Or are they merely mentioned in the text? If so, in depth or in passing? And consequently, how can they be mobilised in a relevant manner to draw such a catalogue? Overall, this comment raises the issues of corpus building (e.g. why not be interested in all research practices in AHSS and only then discriminating which can be deemed open, rather than relying on declarations of openness?) and the limits of corpus analysis (e.g. how to extract actual practices from a non-dedicated corpus?) which are both addressed in the conclusion but possibly in too little details.*

 Given the scope of the exercise attempted here, we felt a literature review focused on intersections of disciplinary discourses with those of openness was appropriate as a way of gaining a broad and wide-ranging sense of how openness is interpreted and deployed across AHSS disciplines. In fact, much of the literature identified was focused on specific practices as manifested in particular disciplines and research types, rather than critiques of open science in general as the reviewer suggests. We hope that sharing the unannotated bibliography of the material returned from these searches (as per another of the reviewer’s comments) will give a greater sense of the rich range of material returned. We would also like to clarify that, as the article notes, open practices provisionally identified among AHSS disciplines from the literature review were subject to substantial further, focused desk research before their suitability for inclusion in the catalogue was determined. Finally, it should be noted that this work formed an initial stage of the MORPHSS project, allowing a broad sense of a landscape of OR in AHSS to emerge; subsequent stages of the project will use qualitative methods e.g. interviews to gain deep understanding of specific practices and contexts located within this landscape.

*Lastly, but beyond the scope of this article, I wonder about the STEM origins of dominant open science regulatory frameworks: although I agree with the authors that these frameworks are focused on narrowly quantitative and positivist epistemologies, do these really come from STEM? Or rather from institutional actors who have a very simple understanding of research practices in STEM reduced to formal procedures, and consequently write policies or design infrastructure on these premises? Works from Leonelli, for example, show how notions of replicability are heatedly debated even within some subdisciplines of the STEM, which would argue for the fact that AHSS are not fundamentally different and, rather, highlight fundamental epistemic issues that also feature in STEM (although less saliently due to the fact that they were historically more formalised over the past two centuries). Beyond the scope of this article, a more detailed sociological analysis of the circulation of such an abstract epistemology, and*

 We agree that this would be a fascinating set of questions to explore in future work. Unfortunately, as noted, it is beyond the scope of the present article.

# Reviewer report: Reviewer response for version 1

**DOI:** 10.5256/f1000research.196728.r476056

**Published:** 2026-05-04

**Authors:** Beatriz Barrocas Ferreira, Maria Manuel Borges

**Affiliations:** 1 Tilburg University University Library, Tilburg, North Brabant, The Netherlands; 2 University of Coimbra, Coimbra, Portugal; 3 Centre for Interdisciplinary Studies - CEIS20, University of Coimbra Center for the Study of Law on Planning Urbanism and the Environment, Coimbra, Coimbra District, Portugal

The manuscript addresses a question that is both conceptually and politically significant: how open research can be understood beyond dominant Open Science frameworks that have largely been shaped around STEM assumptions, particularly around reproducibility, data sharing, and technical transparency. It is a very strong, timely, and genuinely important contribution to current debates on open research in the arts, humanities, and social sciences.

 The authors aim to rethink openness in ways that are more sensitive to the epistemic traditions and values of AHSS. To do so, they combine a literature-based mapping exercise with the development of the MORPHSS typology, identifying six forms of openness organised across three broader categories: openness as equitable and inclusive knowledge creation, openness as the rich realisation of research, and openness as access/ible dissemination. The manuscript also offers reflections and recommendations for funders, institutions, publishers and researchers, arguing for a more inclusive and situated understanding of open research.

 Overall, this is an excellent manuscript. It is theoretically strong, methodologically rigorous, and well written, and makes an important intervention in a landscape where discussions of openness still too often default to STEM-based assumptions. One of its main strengths is that it does not simply argue for the inclusion of humanities practices within existing Open Science models but instead challenges the adequacy of those models themselves.

 The comments below are therefore intended as suggestions for strengthening what is already a very robust article. They are mostly conceptual rather than corrective. We would therefore like to leave it to the authors to take them into consideration or discard them.

**Main comments**

**1. Conceptual breadth of Open Research**

 The manuscript’s broad and inclusive understanding of openness is one of its greatest strengths, particularly in showing how dominant definitions of Open Science have often been too narrow to account for the epistemic traditions of AHSS. At the same time, this conceptual expansion could benefit from a slightly clearer reflection on the boundaries of the term itself. The manuscript brings together several practices under the umbrella of open research, but this can also challenge conventional assumptions of best practices, raising the question of how openness is distinguished from broader notions of good scholarly practice.

 A more explicit clarification of where the authors see the conceptual boundaries of openness, and how openness is distinguished from broader notions of good scholarly practice, could strengthen the analytical precision of the AHSS framework without reducing its inclusiveness.

 2. **Recognition of existing humanities practices versus translation into Open Science vocabulary**

 Related to the previous point, the manuscript could reflect more explicitly on whether it is identifying "genuinely" open practices, or whether it is retrospectively translating long-established humanities and qualitative research traditions into the language of Open Research.

 Practices such as reflexivity, participatory methods, collaborative authorship, and public scholarship did not emerge because of Open Research, and in many cases, they predate current open research agendas. The manuscript rightly highlights this, but we think the implications deserve stronger discussion.

 This raises the question whether the goal is to recognise that humanities fields have always practiced forms of openness that policy frameworks have failed to acknowledge, or if translating these practices into open research language risk subordinating them to policy frameworks they did not originate from. 

 3. **Situated openness and institutional evaluation regimes**

 One of the most important arguments in the manuscript is that openness in AHSS is often situated, contextual and resistant to binary measurement. This is particularly convincing in relation to participatory research, qualitative data sharing, CARE principles, and reflexivity.

 At the same time, universities, funders and assessment systems increasingly operate through indicators, compliance mechanisms and measurable outputs. This creates an important tension that we think could be explored more directly, namely on how can situated openness survive within institutional systems that depend on standardisation.

 The recommendations section touches on this, but a deeper reflection on the political and institutional implications could strengthen the argument. Additionally, the discussion of monitoring initiatives such as the French Open Science Monitor and the UKRN Open Research Indicators Project is important. We would suggest expanding this slightly, as it is one of the clearest examples of the tension between institutional governance and situated openness.

 If open research is increasingly governed through funding mandates, monitoring systems, and evaluation frameworks, there is a risk that even participatory and epistemically inclusive forms of openness become reduced to another form of compliance.

 4. **Distinguishing accessibility from democratisation**

 The distinction between “availability of outputs” and “accessible communication of research” is very useful. However, it may also be helpful to make clearer the distinction between accessibility and democratisation.

 For example, making research available through podcasts, exhibitions or public scholarship certainly improves access and engagement, but it does not necessarily redistribute epistemic authority or participation in knowledge production.

 Since the manuscript makes strong claims about openness as equitable and inclusive knowledge creation, clarifying this distinction could strengthen the conceptual coherence of the framework.

**Minor suggestions**

- The MORPHSS typology of forms of openness in AHSS is excellent and likely to become one of the manuscript’s most cited contributions. We wonder whether a short paragraph reflecting on how the typology might evolve over time, or where its boundaries remain intentionally open, could strengthen its long-term usefulness
- A minor editorial point: the reference list does not appear to be fully organised in alphabetical order and would benefit from a final check for consistency

**Final remarks**

 Overall, we found this to be an excellent manuscript and a substantial, timely contribution to discussions of open research in the humanities and social sciences, both conceptually and practically. Our suggestions are intended only to help strengthen an already strong article and to sharpen some of the conceptual tensions that make it so relevant. We would also like to congratulate the authors on the quality of the work and on conducting such important research, which will be valuable not only for scholars working in AHSS, but also for broader debates on the future of open research and scholarly communication.

Is the work clearly and accurately presented and does it cite the current literature?

Yes

If applicable, is the statistical analysis and its interpretation appropriate?

Not applicable

Are all the source data underlying the results available to ensure full reproducibility?

No source data required

Is the study design appropriate and is the work technically sound?

Yes

Are the conclusions drawn adequately supported by the results?

Yes

Are sufficient details of methods and analysis provided to allow replication by others?

Yes

Reviewer Expertise:

Scholarly communication, open research, critical librarianship, academic libraries

We confirm that we have read this submission and believe that we have an appropriate level of expertise to confirm that it is of an acceptable scientific standard.

# Response

**Published:** 2026-08-07

**Authors:** Jenni Adams

**Affiliations:** The University of Sheffield Information School, Sheffield, England, UK

*Conceptual breadth of Open Research. The manuscript’s broad and inclusive understanding of openness is one of its greatest strengths, particularly in showing how dominant definitions of Open Science have often been too narrow to account for the epistemic traditions of AHSS. At the same time, this conceptual expansion could benefit from a slightly clearer reflection on the boundaries of the term itself. The manuscript brings together several practices under the umbrella of open research, but this can also challenge conventional assumptions of best practices, raising the question of how openness is distinguished from broader notions of good scholarly practice. A more explicit clarification of where the authors see the conceptual boundaries of openness, and how openness is distinguished from broader notions of good scholarly practice, could strengthen the analytical precision of the AHSS framework without reducing its inclusiveness.*

 We feel that this comment is in large part aimed at the issues raised and responded to in the subsequent comment - namely, the extent to which open research is about practices that form part of existing disciplinary traditions, or new and emergent practices introduced as part of a self-consciously ‘open’ agenda. In terms of the conceptual boundaries of openness, we feel the MORPHSS typology speaks to this question by offering a detailed characterization of the dimensions of openness as we conceive them.

*Recognition of existing humanities practices versus translation into Open Science vocabulary. Related to the previous point, the manuscript could reflect more explicitly on whether it is identifying "genuinely" open practices, or whether it is retrospectively translating long-established humanities and qualitative research traditions into the language of Open Research. Practices such as reflexivity, participatory methods, collaborative authorship, and public scholarship did not emerge because of Open Research, and in many cases, they predate current open research agendas. The manuscript rightly highlights this, but we think the implications deserve stronger discussion. This raises the question whether the goal is to recognise that humanities fields have always practiced forms of openness that policy frameworks have failed to acknowledge, or if translating these practices into open research language risk subordinating them to policy frameworks they did not originate from.*

 As the article states, the open research practices we identify among AHSS fields are a combination of practices that predate current open research agendas - but which, we feel, can be meaningfully understood as open practices, and can valuably diversify understandings of the scope and manifestations of open research as a result - and more emergent practices such as Annotation for Transparent Inquiry, pre-registration for qualitative research, and experiments with open peer review via open interaction and participation. We feel this balanced understanding is made clear in the article, which argues for the benefits of such an inclusive approach, in ways that have implications for policy (as the reviewers highlight) but are not reducible to this context.

*Situated openness and institutional evaluation regimes - One of the most important arguments in the manuscript is that openness in AHSS is often situated, contextual and resistant to binary measurement. This is particularly convincing in relation to participatory research, qualitative data sharing, CARE principles, and reflexivity. At the same time, universities, funders and assessment systems increasingly operate through indicators, compliance mechanisms and measurable outputs. This creates an important tension that we think could be explored more directly, namely on how can situated openness survive within institutional systems that depend on standardisation. The recommendations section touches on this, but a deeper reflection on the political and institutional implications could strengthen the argument. Additionally, the discussion of monitoring initiatives such as the French Open Science Monitor and the UKRN Open Research Indicators Project is important. We would suggest expanding this slightly, as it is one of the clearest examples of the tension between institutional governance and situated openness. If open research is increasingly governed through funding mandates, monitoring systems, and evaluation frameworks, there is a risk that even participatory and epistemically inclusive forms of openness become reduced to another form of compliance.*

 We agree that the tension between situated openness and the institutional dependency on standardisation (and automation) to survive is important to consider. We have added a brief section to the ‘Recommendations’ section of the article to highlight this tension and the need for modified approaches to open research evaluation in response. We feel these questions might also valuably inform a more substantial future piece of work around the implications of situated openness for efforts towards research evaluation.

*Distinguishing accessibility from democratisation - The distinction between “availability of outputs” and “accessible communication of research” is very useful. However, it may also be helpful to make clearer the distinction between accessibility and democratisation. For example, making research available through podcasts, exhibitions or public scholarship certainly improves access and engagement, but it does not necessarily redistribute epistemic authority or participation in knowledge production. Since the manuscript makes strong claims about openness as equitable and inclusive knowledge creation, clarifying this distinction could strengthen the conceptual coherence of the framework.*

 Democratisation is not a concept the article depends heavily on, with just one reference in the main body of the article that is situated in a section of literature review. We have added a caveat to this usage to make clear the lack of straightforward equation between accessible communication and democratisation.

*The MORPHSS typology of forms of openness in AHSS is excellent and likely to become one of the manuscript’s most cited contributions. We wonder whether a short paragraph reflecting on how the typology might evolve over time, or where its boundaries remain intentionally open, could strengthen its long-term usefulness*

 We appreciate this comment about the MORPHSS typology of openness. The Catalogue of Open Practices is an evolving resource which we will continue to develop over the course of the project, reflecting iteratively on the typology of openness as we do so. We are already taking steps to augment the catalogue, taking on board suggestions from different external contributors. Given that this process is difficult to anticipate, we have chosen not to attempt to do so within the boundaries of the article.

*A minor editorial point: the reference list does not appear to be fully organised in alphabetical order and would benefit from a final check for consistency*

 We have made a small number of corrections to the alphabetical sequencing of references.
